# Estudio De La Vida Bajo Estres: Methodological Overview and Baseline Data Analysis of a Case-Control Investigation of Risk and Resiliency Factors for Traumatic Stress in Colombia

**DOI:** 10.1007/s10862-025-10203-1

**Published:** 2025-03-01

**Authors:** M. Robinson, E. McGlinchey, Y. Ardila, F. Guillen, N. Acosta, J. Gomez, NI. Bloch, D. Hanna, V. Akle, C. Armour

**Affiliations:** 1 Research Centre for Stress Trauma and Related Conditions (STARC), School of Psychology, Queen’s University Belfast, David Keir Building, 18-30 Malone Road, Belfast, BT9 5BN, Northern Ireland, UK; 2 School of Medicine, Universidad de Los Andes, Bogota, Colombia; 3 Department of Biomedical Engineering, Universidad de Los Andes, Bogota, Colombia; 4 Center for Clinical and Translational Research, La Misericordia Clínica Internacional, Barranquilla, Colombia; 5 Facultad de Ciencias de la Salud, Universidad Simon Bolivar, Carrera 54 No 64-222, Barranquilla, Colombia; 6 Instituto Cardiovascular del Cesar, Valledupar, Colombia

**Keywords:** Posttraumatic stress disorder, Biopsychosocial, Risk factors, Resilience factors, Longitudinal, Colombia

## Abstract

The *Estudio de la Vida Bajo Estres* (My Life Under Stress [MI-VIDA] Study) aims to investigate risk and resilience factors associated with the development of PTSD in a trauma-exposed sample of more than 500 Colombian residents exposed to this country’s armed conflict. The study utilised a longitudinal case-control design capturing psychosocial data over 18 months, in addition to baseline DNA samples for a parallel genomic analysis. This paper specifically provides an overview of the design and methodology of the wider investigation, and reports baseline characteristics including sociodemographic information and mental health outcome prevalences from this hard-to-reach and under-researched population. Results of baseline analysis suggested that one third (34.88%) of this trauma-exposed sample screened positively for PTSD. Participants endorsed high numbers of potentially traumatic experiences including Forced Displacement (88.61%), Exposure to Severe Human Suffering (53.91%), Combat Exposure (53.02%), and Physical Assault (51.78%). Participants also reported relatively high levels of mental ill-health including depression (29.90%) and anxiety (27.56%). The number of traumas experienced, and the reported comorbid difficulties were generally higher among those who screened positive for PTSD. These preliminary analyses detail the baseline characteristics, and the relative burden of mental ill-health in this trauma-exposed sample. The wider study comprising longitudinal measurement of these conditions has the potential to make a significant contribution to the understanding of risk and resiliency factors for posttraumatic stress in this unique Latin American context.

**Supplementary Information:**

The online version contains supplementary material available at 10.1007/s10862-025-10203-1.

## Introduction

According to the registry of victims in Colombia over nine million people have been affected by the armed conflict between the Colombian government and the FARC-EP (*Fuerzas Armadas Revolucionarias de Colombia “Ejército del Pueblo”)* [Revolutionary Armed Forces of Colombia – People´s Army]) guerrillas over the past 50 years. The Colombian government and guerrillas most recently agreed a ceasefire in September 2016 in the hopes of moving forward with peace negotiations. As a result of the peace accord, the FARC pledged to disarm and reincorporate into civilian society while the government has vowed to help former rebels and the wider population through a special economic program, the PDET (“Programa de Desarrollo con Enfoque Territorial”, [Development Programs with Territorial Focus]). This program has the goal of creating inclusive economic opportunities prioritising residents of rural communities who have been harmed by the conflict.

This approach suggests that peacebuilding efforts must go beyond the cessation of violence to include building a new sense of identity and citizenship to foster change at all levels of community organisation (Escobar Arango, 2017). Within the goals of the PDET programme, the government has committed to promoting mental health and welfare in the affected communities recognising the health burden and service disruption in these areas. Indeed, this has been highlighted as a serious health concern with up to 40% of the Colombian population experiencing a mental health disorder in their lifetime (Ministerio de la Protección Social, 2005) exacerbated by the relative impacts of traumatic stress and restricted access to services (Giebel et al., 2022).

Evidence from the World Health Organization (WHO), World Mental Health Surveys on the burden of psychological ill-health in Colombia, has reported a relatively high rate of trauma exposure (82.7%) at the population level (Koenen et al., 2017). Epidemiological evidence has further suggested that lifetime prevalence in the region for depression or anxiety is 10.1% (Gomez-Restrepo et al., 2016) and lifetime Posttraumatic Stress Disorder (PTSD) prevalence rates between 1.8 and 6.7% (Koenen et al., 2017). It has been postulated that those living in rural areas may be more likely to be exposed to traumatic stressors such as conflict and violence giving rise to differences in mental health outcomes (Gaviria et al., 2016). Furthermore, emergent evidence has suggested that prevalence rates of mental ill-health have markedly increased from 2020 (Martínez-Cabezas et al., 2023), which is unfortunately a global trend following the COVID-19 pandemic (COVID-Mental Disorders Collaborators, 2021). Taking this evidence into consideration, there is a clear and critical need to improve our understanding of risk and resiliency factors that influence the development of mental health disorders in the region and in Global South countries in general (Hinton & Lewis-Fernandez, 2011; Lund et al., 2018).

Current evidence highlights several risk factors for the development of PTSD that have been robustly observed globally including: economic inequality and rurality (McCall-Hosenfeld et al., 2014), conflict exposure (Charlson et al., 2016), and community violence (Fowler et al., 2009). Notably the combined experience of conflict, violence, and socioeconomic inequality is argued to place affected groups in Colombia at increased risk for PTSD (Botero-Rodríguez et al., 2021). Additionally, over 4.8 million people have been internally displaced in Colombia (Internal Displacement Monitoring Centre, 2023); with displacement being a recognised risk factor for PTSD (Morales Mesa et al., 2021).

Globally there are a myriad of reported psychosocial factors robustly evidenced as risks associated with traumatic stress in adult samples. Indeed, an extensive meta-analytic study reported that age, gender, and psychiatric comorbidity are repeatedly found amongst a broader list of associated risk factors for traumatic stress outcomes (Brewin et al., 2000). This is a particularly relevant consideration in Low and Middle Income Countries (LMIC’s), and those affected by conflict, where there is noted to be additional contextual risk and greater burden of mental ill-health associated with resource limitation exposure to traumagenic environments, those in which one has a higher likelihood of trauma exposure (Hoppen et al., 2021). Prior evidence suggests that no one risk factor is deterministic of traumatic stress outcomes, however the cumulative burden of trauma exposure and multiple risk factors may confer additive risk for mental ill-health (Brewin et al., 2000; Breslau et al., 2008). Evidence is however critically lacking in Latin American contexts, and there remains a need to understand the contributing role of demography and psychosocial variables as risk and/or resiliency factors for traumatic stress outcomes in post-conflict contexts in the region.

Likewise, there is evidence that genetic ancestry and background could influence the risk of developing PTSD (Kremen et al., 2012; Nievergelt et al., 2019). There are suggested to be complex interactions of environmental and genetic factors which influence the likelihood one may experience PTSD (Zannas et al., 2015); however, this research is mostly limited to Western countries (Hinton & Lewis-Fernandez, 2011). A large meta-analysis of genome wide association studies including over 27,000 people with PTSD evidenced high overlap between genes associated with PTSD and other related psychiatric disorders such as major depressive disorder, as well as some loci that are uniquely associated with PTSD (Nievergelt et al., 2019; Stein et al., 2021). Studying the genetic underpinning of psychiatric disorders in concert with psychosocial influences is a crucial step to thoroughly understanding their causes and treatment strategies. Therefore, any comprehensive investigation of mental health risk and resilience factors should consider biological/ genetic factors in addition to psychosocial factors. However, the expression of the genetic factors underlying PTSD is strongly dependent on genetic background (Nievergelt et al., 2019) and poorly studied beyond populations in developed countries.

Further to this, individuals who are exposed to repeated or ongoing traumatic stress and adversity may be at unique risk for psychopathological outcomes (Greene et al., 2018). In such contexts, reactions typically categorised as pathological may be considered adaptive; for instance arousal and avoidance of trauma reminders may serve to protect the individual from viable environmental threats (Diamond et al., 2010). Those in Colombia have been affected by an extended period of conflict trauma, community violence, and displacement which has contributed to a significant mental health burden in the region, particularly those areas most affected by ongoing community-level stressors (Cuartas & Roy, 2019; Gaviria et al., 2016; Tamayo-Agudelo & Bell, 2019).

There is a need to understand the influence of these diverse risk factors over time (Zannas et al., 2015), and in the specific context of Colombia where evidence is lacking. This study therefore adopted a longitudinal design assessing a battery of evidenced risk and resilience factors at three timepoints over 18 months. In addition, biological samples (saliva) were provided by all participants at baseline. Combining these two modes of data collection the goal of the wider investigation was to build a biopsychosocial model of risk of a PTSD outcome after trauma exposure in this uniquely affected sample. The primary aim of this paper is to provide a methodological overview of this novel investigation, detailing the assessment and procedures implemented with a focus on the core psychosocial data collection in the MI-VIDA [My Life] investigation (i.e., assessment of lifetime trauma experiences and current mental ill-health), and present descriptive information captured at baseline to contextualise this research and sample. Data related to biological sampling will be forthcoming in future publication.

## Methodology

### Design

The MI-VIDA study is a case-control cohort investigation of biopsychosocial risk and resiliency factors. This observational study design sought to assess differences between trauma exposed participants who screen positive for probable PTSD (Case group) with those who do not (Control group) identifying potential salient factors in the development of this condition. Participants were eligible for enrolment in this study if they endorsed a trauma exposure during their lifetime. Groups were divided by categorisation of probable PTSD diagnosis based on an empirically supported cut-off score of 33 on a self-report measure of PTSD, the PTSD Checklist for DSM-5 (Murphy et al., 2017; National Centre for PTSD, 2022; Weathers et al., 2013a, b). This screening criteria is similarly used in previous studies of PTSD in Colombia (see Botero-Rodríguez et al., 2021).

### Population

The population of interest were trauma-exposed Colombian Adults (aged + 18), most of them living in the proximity of Valledupar (Department of Cesar) and other municipalities of the Sierra Nevada de SantaMarta; Serrania del Perijá, and Zona Bananera PDET, and from Barranquilla (Department of Atlantico). These areas were selected for recruitment as they have historically experienced heightened rates of conflict and violence (Jiménez Ortega et al., 2019), and thus considered an area of particular need. Individuals were recruited to this study through promotion of the study through community leaders and advertisement in healthcare settings. Through participatory consultation with community leaders this recruitment strategy was determined as appropriate for the current investigation to facilitate engagement with this group previously marginalised by academic research. All were provided travel and nominal time compensation for their participation as to not economically disadvantage those who took part.

A-priori sample size calculations suggested *N* = 398 would be optimal for powered analyses (*f*^2^ = 0.04, 95% confidence interval, 0.80 power) in multivariate regression models with up to nine predictor variables. Accounting for potential non-completion and attrition this study sought to recruit up to *N* = 600 participants providing accompanying biological data prior to stratification into Case and Control groups to ensure adequate sample size. This decision was made pragmatically; in longitudinal investigations of traumatic stress it is noted that retention rates may be lowered by participant tendency for avoidance, reduced personal mobility, and functional impairment among those experiencing distress (Scott et al., 2006).

### Procedure

Study participants were surveyed with repeated measures; three surveys with approximately eight months between administrations. Initial data collection, i.e. baseline survey administration, began in February 2022 and was completed in June 2023. Participants were asked to complete survey measures in a self-report format or facilitated by a Spanish-speaking volunteer research assistant to read questions and record participant responses. Surveys were completed within recruitment events at locations with appropriate physical and technological resources to support study administration; e.g. healthcare clinics (Instituto Cardiovascular del Cesar, La Miscordia Clínica International), universities (Universidad Simón Bolivar, Fundación Universitaria del Área Andina), and local libraries.

All data were collected using an electronic survey administered through the REDCap platform hosted by La Misericordia Clínica International (Harris et al., 2019). All participants were presented a comprehensive participant information sheet in Latin American Spanish. This detailed the study procedure at Time 1, including the collection of biological samples, and expected schedule for recontact to complete psychosocial surveys at follow-up timepoints. Potential respondents were eligible to participate if they were aged 18 or over, reported experience of at least one lifetime stressor, and confirmed they were not experiencing a current mental health or learning difficulty that would affect their ability to provide informed consent. Participants then provided informed consent confirming their understanding of the study requirements and to be recontacted at follow-up timepoints.

During Baseline data collection participants were also asked to provide a 2 ml saliva sample in accordance with the collection kit manufacturer instructions (IsoHelix, 2021). These samples were labelled and archived for genomic analysis.

At baseline data collection, participants were offered an incentive of 25,000$COP (approx. $6.20 USD) and compensated for travel costs upon presentation of a receipt. At follow-up timepoints participants were offered the opportunity to complete survey measures over the phone with a Clinical Psychologist on the study or to return to the study site. Participants were offered the same compensation for their travel and time at follow-up.

### Translation

The researchers attempted to identify Latin American Spanish translations of relevant measures within the survey. Where instruments were not available in Spanish (see Supplementary Table 1) a process of *translation-back-translation* was used to ensure cross-cultural equivalence of the survey measures. This process involves the translation of original survey measures (English) to the target language (Latin American Spanish) by one translator, followed by translation of the target version back to the original language by another translator (Maneesriwongul & Dixon, 2004) to ensure consistency of content and meaning (Brislin, 1970). All measures were translated and back-translated by bilingual (English & Spanish speaking) members of the research team independently, and concordance reviewed by an English-speaking researcher experienced in psychometric administration. Informed consent and instructional materials accompanying survey measures were subject to the same translation procedure.

The Spanish translated surveys were reviewed, commented on, and validated by “Unidad para las Víctimas” [Unit for Victims] community leaders in a focus group. This step was taken to confirm the that translated measures were understood by local representatives in the current study context, and that their administration was seen as appropriate and relevant. The results of this consultation did not highlight any issues with administration of these survey measures.

### Included Measures

This study included a wide range of self-report measurements across timepoints. The psycho-social measures administered across this study, including at which timepoints they were administered and detailing of which were translated for the current investigation, are summarised in Supplementary Tables 1, and detailed below.

*Demographic information* was collected using a bespoke battery of items capturing basic descriptive information (e.g. gender, age, marital status, household income). Based on previous literature this section also captured information on living area (*Urban*, *Rural*), and related to victim registry status thought to be relevant to risk factors for trauma exposure and traumatic stress outcomes. Participants were asked if they had previously registered with the “Unidad para las Víctimas” ([Unit for Victims], a governmental body that aims to recognise and support those effected by the armed conflict in Colombia) and if they considered themselves to have been a victim of the armed conflict but had not formally registered their victim status with the governmental organisation.

### Traumatic Life Events and Appraisals

*Exposure to potentially traumatic life events* was measured using an adaptation of the Life Events Checklist for the Diagnostic and Statistical Manual 5 (LEC-5; Weathers et al., 2013a, b). The LEC-5 is a 17-item screening measure of stressful life events aligned with the DSM-5 definition of potentially traumatic life events qualifying as antecedents for PTSD diagnosis. Four additional items were added to this screener judged to be potentially relevant to the study population; exposure to physical torture, psychological torture, property damage, and forced displacement (see Taylor, 2016). Responses to all items were binary endorsements (“*Yes*”, “*No*”) and any positive trauma endorsements further queried if they related to the armed conflict in Colombia (“*Yes*”, “*No*”). The LEC-5 and tailored versions thereof have been widely used to assess exposure to potentially traumatic life events, including in Colombia (Gray et al., 2004; Guillen-Burgos et al., 2023).

*Exposure to adversity in childhood* was measured using the Adverse Childhood Experiences questionnaire (ACE; Felitti et al., 1998). The ACE is a 10-item, widely used and validated measure of adverse childhood experiences, which are retrospectively recalled among adult respondents. Reponses are binary coded (“*Yes*”, “*No*”) with scores assigned 1 and 0 respectively for each item. An aggregated score of adversity is calculated by summing all responses with greater scores indicative of more adverse experience in childhood. The current study administered a translated version of this measure previously used in Colombia (Von Calero, 2016).

*Exposure to anti-social behaviour* was measured using a single item querying exposure to a number of potentially stressful events within one’s community in the previous five years (e.g. murder, extortion, sexual harassment, street fights). This measure was adopted from a previous study examining the influence of current community threat on mental ill-health in Colombia (Taylor, 2016). Respondents are asked to select all events that they have experienced in the previous five years, thus the number of events endorsed providing an indication of greater political violence and conflict-related stress exposure (Taylor, 2016).

*Appraisal of traumatic life events* was measured using a translated Spanish language version of the Trauma Appraisal Questionnaire (TAQ; DePrince et al., 2010). The TAQ consists of 54 items asking respondents to reflect on how they feel currently in relation to exposure to an elected potentially traumatic event. These items align to six latent factors capturing categories of emotion; Betrayal, Self-blame, Fear, Alienation, Anger, and Shame (DePrince et al., 2010). Responses are coded on a 5-point Likert scale ranging from 1 “*Strongly disagree*” to 5 “*Strongly agree*”, and a mean score is calculated for items related to each subscale providing an indicator of these appraisal styles. The TAQ has previously been validated as a measure of trauma-related cognitions among community samples and has shown good psychometric properties across administrations (DePrince et al., 2010; Gomez de La Cuesta et al., 2019).

### Psychological Risk/Resiliency Factors

*Psychological coping strategies* were measured using the Spanish language version of the Brief COPE questionnaire (Perczek et al., 2000). The Brief COPE is a 28-item measure of coping styles in response to stressful situations in the previous year (Carver, 1997). Reponses are coded on a 4-point Likert scale with participants reflecting on prior coping behaviours; 1 “*I didn’t do this at all*” to 4 “*I did this a lot*”. This measure provides an indicator of 14 coping styles (e.g. Active, Planning, Self-Distraction, Behavioural Disengagement) by summing scores for two items related to each coping style (Carver, 1997). The Brief COPE has been widely validated as a measure of psychosocial coping, including in trauma-exposed (Wang et al., 2018) and Spanish-speaking populations (Fernandez-Martin et al., 2022; Perczek et al., 2000).

*Coping Flexibility* was measured using the Coping Flexibility Scale Revised (CFS-R; Kato, 2012). The CFS-R comprises 12-items querying individuals typical response to stress coping with each response rated on a 4-point Likert scale from 0 “*Not applicable*” to 3 “*Very applicable*”. This measure may be used to compute a total coping flexibility score or summed using three subscales; strategy abandonment, re-coping, and meta-coping. This scale has been demonstrated to have acceptable psychometric properties, and be robustly associated with conceptually related constructs suggesting it’s convergent validity (Kato, 2020).

*Trait psychological resilience* was measured using the Spanish Language version of the Connor-Davidson Resilience Scale (CD-RISC; Connor & Davidson, 2003). The current study administered the 10-item version of the scale which assessed resilience as unidimensional construct. Reponses are reported on a 5-point Likert scale from 0 “*Not true at all*” to 4 “T*rue nearly all the time*”. Scores on this scale are summed with higher scores indicative of great trait resilience. The psychometric properties of this scale have been validated previously in trauma exposed samples (see Karairmak, 2010), and in Spanish-language administrations (see Notario-Pacheco et al., 2011).

*Perceived meaning in life* was measured using the Spanish version of the Meaning In Life Questionnaire (MIL; Marco et al., 2022; Steger et al., 2006). The Spanish language version has been previously validated (Marco et al., 2022). This 10-item measure assesses presence and search for meaning in one’s life where respondents reflect how true statements reflect their beliefs. Responses are recorded on a 7-point Likert scale ranging from 1 “*Absolutely true*” to 7 “*Absolutely untrue*”, with scores summed for the presence of *meaning* and *search for meaning* subscales with higher scores indicative of greater perceived meaning in life. This is noted to be the most widely used measure of perceived meaning in life, and has been adequately validated in terms of reliability and validity of the measure’s Spanish-language version (Marco et al., 2022).

*Optimism* was measured using the State Optimism Measure (SOM; Millstein et al., 2019). This 7-item measure assesses self-perceived optimism about future events. Each response is rated on a 5-point Likert scale from 1 “*Strongly disagree*” to 5 “*Strongly agree*” and summed to provide a univariate indication of current optimism with higher scores indicative of more optimistic beliefs. In initial validation this measure has demonstrated good reliability and validity among community and medical recovery populations (Millstein et al., 2019).

*Happiness* was assessed using a single self-report item with respondents rating general happiness on a scale from 0 to 10 with greater scores indicative of greater perceived general happiness. This has been previously demonstrated to be a psychometrically valid and reliable measure of state happiness and related to general health and wellbeing (Abdel-Khalek, 2006).

*Perceived deservedness of persecution* was measured using the Spanish language version of the Perceived Persecution and Deservedness Scale (PaDS; Melo et al., 2009). The PaDS is a 10-item measure of paranoia (e.g. “There are times when I worry that others might be plotting against me”) and persecutory thinking (e.g. “People will almost certainly lie to me”). Reponses are recorded on a five-point Likert scale from “Not at all” to “Very much”. Where respondents endorse any of these items they are ask to what extent they believe they deserve to feel this was on the same Likert scale. Summary scores for Persecution and Deservedness subscales are obtained by computing a mean for relevant items with higher scores indicative of greater perceived persecution and deservedness respectively.

*Loneliness* was measured using the brief adaptation of the UCLA Loneliness Scale (Hughes et al., 2004). This three-item measure specifically developed to enable large-scale survey research assesses perceived loneliness as a univariate construct with typical feelings of loneliness recorded on a scale from 1 “*Hardly Ever*” to 3 “*Often*”. Response scores are summed to provide an overall measure with greater scores indicative of greater feelings of loneliness. Review of the UCLA Loneliness Scale administration suggests this measure to have excellent psychometric properties, and moderate-high quality evidence in support of this (Alsubheen et al., 2021).

*Emotional Regulation* was measured using the Difficulties in Emotional Regulation Scale Short Form (DERS-SF; Kaufman et al., 2015). The DERS-SF is an 18-item measure of affect regulation across six sub-domains; Nonacceptance of emotional responses, difficulty engaging in goal-directed behaviour, impulse control difficulties, lack of emotional awareness, limited access to emotion regulation strategies, and lack of emotional clarity. Items query how often difficulties in affect regulation are generally experienced from 1 “*Almost never*” to 5 “*Almost always*”. Responses are summed for each subscale and for the total measure with greater scores indicative of difficulties in emotion regulation. The psychometric properties of this scale have been evidence in community and clinical populations (Hallion et al., 2018; Kaufman et al., 2015).

*Cognitive Flexibility * (i.e. the ability, willingness, and self-efficacy to adapt in response to contextual cues) was measured using the Cognitive Flexibility Scale (CFS; Martin & Rubin, 2016). The CFS is composed of 12 items rating agreement with statements about typical beliefs and feelings on a six-point Likert scale from 1 “*Strongly disagree*” to 6 “*Strongly agree*”. Scores are summed to provide a global score of cognitive flexibility. In initial development the validity of this scale was validated in three non-treatment seeking samples, and shown to be associated with assessment of concurrent constructs and with communication efficacy and ratings form others (Martin & Anderson, 1998; Martin & Rubin, 2016).

### Social Risk/Resiliency Factors

*Perceived social support* was measured using the Spanish language version of the Multidimensional Scale of Perceived Social Support (MSPSS; Cobb & Xie, 2015; Zimet et al., 1988). The MSPSS is a 12-item measure of social support in three domains; from family, from friends, and from one’s significant other. Responses are recorded on a 7-point Likert scale from 1 “*Very strongly disagree*” to 7 “*Very strongly agree*”, with scores summed to provide a measure of overall perceived social support, and perceived support in each domain. This measure has been shown to be a reliable and valid measure of perceived social support (Dambi et al., 2018), including among non-treatment seeking respondents in Colombia (Trejos-Herrera et al., 2018).

*Perceived social trust* was measured using a scale adopted from the Latin American World Values Survey (World Values Survey Association, 2018). This measure consists of 7 items, the first asking if they believe generally “*Most people can be trusted*” or “*You need to be very careful when dealing with people*”. The remaining six items assess to what extent the respondent believes people of different groups (e.g. family, neighbours, people of another nationality) may be trusted on a 4-point Likert scale from 0 “*Do not trust at all*” to 4 “*Trust a lot*”. Items are summed to produce an aggregate measure of social trust with greater scores indicative of broader trust of others across domains (Taylor, 2016). This scale has previously been used as a measure of civic trust correlated with mental ill-health in Colombia (Taylor, 2016).

*Self-reported identity* was measured using the Social and Personal Identity Scale (SPIS; Nario-Redmond et al., 2004). The SPIS assesses to what extent respondents’ importance ascribed to a personal sense of self and in-group identity. This measure consists of 16 items assessing the importance of different aspects of identity from 1 “*Not at all important to who I am*” to 7 “*Extremely important to who I am*”. A mean score is imputed for each subscale providing a score for Personal Identity, and Social Identity. This scale has been shown to possess concurrent and structural validity as a measure of perceived importance of social and personal identity in a Latin American population suggesting this scale to reliably measure these domains (Gaete Fiscella et al., 2023).

*Social desirability bias* was measured using two scales at different timepoints: the Latin American Spanish version of the Marlowe-Crowne Social Desirability Scale – Short Form (MCSD-SF; Cosentino, 2008; Reynolds, 1982), and the Eyesenk Lie Scale (ELS; Eysenck & Eysenck, 1992). The purpose of both measures is to assess the likelihood of respondents providing socially desirable or satisficing answers: The MCSD-SF consists of 13 dichotomous items scored with values of 1 or 0 for “*True*”/ “*False*” responses respectively. Responses are summed to produce a total score with higher scores indicative of a tendency to respond in a socially desirable way. The ELS is a subscale within the Eysenck Personality Inventory consisting of 12 items with dichotomous (“*Yes*”, “*No*”) responses equal to 1 or 0 respectively. Responses on this scale are summed to provide an overall score with greater values indicative of more socially desirable responding. These measures have demonstrated good psychometric properties, including internal reliability and convergent validity in Latin American samples (Cosentino, 2008; Cosentino & Solano, 2012).

### Mental Health Outcomes

*Posttraumatic Stress Disorder* was assessed using the PTSD Checklist for DSM-5 (PCL-5; Weathers et al., 2013a, b) and the International Trauma Questionnaire (ITQ; Cloitre et al., 2018). The PCL-5 is a 20-item measure of PTSD in accordance with DSM-5 criteria, and the ITQ is an 18-item measure of PTSD in accordance with ICD-11 criteria. Both inventories assess to what extent respondents have been bothered by symptoms in the previous four weeks on a 5-point Likert scale from 0 “*Not at all*” to 4 “*Extremely*”, and have demonstrated good psychometric properties in Latin American samples; PCL-5 (see Torres et al., 2022), ITQ (see Fresno et al., 2023). The current investigation included two additional items to the PCL-5 measuring dissociative symptoms on the same response scale to assess the presence of Dissociative Subtype PTSD, a practice adopted by previous research (Ross & Armour, 2022). The PCL-5 was used as the screening measure of PTSD in the current study: core PCL-5 responses may summed across all items to provide an overall measure of symptom-related distress with greater scores indicative of more symptom severity, and a total score of 33 or greater indicative of probable PTSD diagnosis (Weathers et al., 2013a, b; Murphy et al., 2017).

*Dissociation* was more broadly measured using the Spanish version of the Dissociative Experiences Scale Taxon (DES-T; Icarán et al., 1996; Waller et al., 1996). The DES-T is an 8-item measure of the degree to which dissociation is experienced in daily life, with responses on a 10-point Likert scale from “*0%*” to “*100%*” of the time at 10% intervals. A total score is provided by computing the mean of all responses with greater scores indicative of more problematic dissociation. This scale has been validated in Spanish-speaking samples, displaying acceptable reliability and convergent validity with other measures of dissociative experiences (Perona-Garcelan et al., 2021).

*Posttraumatic growth* was measured using the Spanish language version of the Post Traumatic Growth Inventory – Short Form (PTGI-SF; Castro et al., 2015). The PTGI-SF is a 10-item measure of the five different domains of potential positive development since experience of a traumatic stressor (Cann et al., 2010). Responses are recorded to different types of change since trauma on a 6-point Likert scale from 0 “*I did not experience this change […]*” to 5 “*I experienced this change to a very great degree […]*”. Responses are summed to provide a total score with greater scores indicative of greater posttraumatic growth. The Spanish-language version of this measure has shown good psychometric properties including internal reliability, structural, and convergent validity (Castro et al., 2015; Garrido-Hernansaiz et al., 2022).

*General wellbeing* was assessed using the Spanish language version of the Warwick-Edinburgh Mental Wellbeing Scale (WEMWBS; Lopez et al., 2013; Tennant et al., 2007). The 7-item version of the scale used in the current study records respondent wellbeing in the previous two weeks on a 5-point Likert scale from 1 “*None of the time*” to 5 “*All of the time*”. Responses are summed to provide an overall rating of subjective wellbeing with greater scores indicative of positive wellbeing. This scale has been validated as a unidimensional measure of perceived wellbeing, and shown good psychometric properties among Spanish-speaking community samples (Castellvi et al., 2014; Lopez et al., 2013).

*Problematic trait anger* was measured using the Spanish language version of the Dimensions of Anger Reactivity scale (DAR; Kannis-Dymand et al., 2019). The DAR is a 5-item measure of anger reactions in the previous four weeks, including experiences and impact on functioning. Responses are coded on a 5-point Likert scale from 1 “*None of the time*” to 5 “*All of the time*”, which are summed to produce a composite score with greater results indicative of problem anger. This scale has demonstrated good psychometric properties in a Spanish-speaking community sample, with good convergent and discriminant validity (Caycho-Rodríguez et al., 2020).

*Depression* and *Anxiety* were measured using the Spanish version of the Patient Health Questionnaire – 9 (PHQ-9; Kroenke et al., 2001; Miranda & Scoppetta, 2018), and the Generalised Anxiety Disorder scale – 7 (GAD-7; Garcia-Campayo et al., 2010; Spitzer et al., 2006) respectively. Both measures record to what extent individuals have experienced symptoms in the previous two weeks. Responses to both scales are recorded on a 4-point Likert scale from 0 “*Not at all*” to 3 “*Nearly every day*”. For both scales a total score is imputed by summing response scores for each scale respectively. A score of 10 or greater is considered indicative of at least moderate severity of psychopathology. These inventories have been widely used and shown good psychometric properties as screening measures for psychopathology in treatment-seeking and community samples (Saunders et al., 2023; Shevlin et al., 2022), and in Latin-American Spanish administration (Cassiani-Miranda et al., 2021; Villarreal-Zegarra et al., 2023).

*Disordered sleep* was measured using three instruments in different survey waves; the Insomnia Severity Index (ISI; Bastien et al., 2001; Fernandez-Mendoza et al., 2012), the Fear of Sleep Inventory (FOSI; Pruiksma et al., 2014), and the Pittsburgh Sleep Quality Index (PSQI; Carpenter & Andrykowski, 1998; Rodriguez-Morales et al., 2018). Responses to the ISI and FOSI represent the degree to which respondent have been bothered by sleep difficulties in the previous two and four weeks respectively on a scale from 0 to 4, where the PSQI responses relate to usual sleep habits without a reference timescale rated on a scale from 0 to 3. For all measures scores are summed across the inventory with greater scores indicative of more disturbed sleep. The ISI and PSQI have previously been validated in Spanish-speaking samples showing good psychometric properties; reliability and convergent validity (Fernandez-Mendoza et al., 2012).

*Substance misuse* was measured using the Spanish Alcohol Use Disorders Identification Test (AUDIT; Ballester et al., 2021). The AUDIT is a 10-item measure of problematic alcohol use with items assessing the frequency of alcohol consumption and dependence on a 5-point Likert scale from 0 “*Never*” to 4 “*Daily or almost daily*”. Responses are summed to provide an overall score, with a score of 10 or greater indicative of problematic alcohol consumption (Babor et al., 2001). This measure version has been shown to possess good psychometric properties and adequately identify problematic alcohol consumption in a Spanish-speaking community sample (Ballester et al., 2021). Additionally, a single item assessment of drug abuse was included to screen for history of illicit substance misuse (Smith et al., 2010).

*Help seeking behaviour* was measured using a translated version of an instrument used in previous studies of traumatic stress and wellbeing (Armour et al., 2021). This measure calls for binary (“*Yes*”, “*No*”) responses to four items assessing if participants have ever taken or currently take medication for a psychological condition, and if participants have ever received or currently receive psychotherapy for a mental health problem.

### Biological Samples and Data

All participants provided salivary samples using the GeneFix DNA saliva collection kit (GFX-02, Isohelix), which stabilizes DNA samples at room temperature for long term storage. The DNA in samples will be isolated, purified, and processed for sequencing using a Infinium PsychArray-24 illumina microarray (Infinium, 2023), which is used for large-scale genetic studies focused on psychiatric predisposition and risk. These arrays have been previously used in an investigation of genomic risk factors for mental ill-health (see Misganaw et al., 2019). The PsychArray-24 BeadChip array includes Single Nucleotide Polymorphisms (SNPs) which have previously been associated with common psychological and psychiatric disorders such as major depressive disorder, obsessive compulsive disorder and with PTSD in particular (see Misganaw et al., 2019).

### Data Analysis

The wider MI-VIDA study aims to examine risk and resiliency factors associated with the development of PTSD and other mental health symptomatology following trauma, however the remit of the current paper is to describe the data structure and methodology at baseline as a whole then subdivided by the Case/ Control qualifier (i.e. probable PTSD as per scores of 33 or greater on the PCL-5). This investigation presents:

- an overview of demographic information of participants in this study by group,
- descriptive statistics related to trauma endorsements by group, and.
- descriptive statistics related to a range of mental health outcomes recorded at baseline.

This overview provides cursory detail of the range of outcome measures included the MI-VIDA investigation, and a core subset of these are used to provide a descriptive overview of the sample in this study. Internal reliability of measures used to quantify mental health outcomes in these descriptive analyses were assessed with Cronbach’s alpha (α) results for these found to be acceptable (see DeVillis, 2016): PTSD measured using the PCL-5 (α = 0.95), Depression measured using the PHQ-9 (α = 0.91), Anxiety measured by the GAD- 7 (α = 0.92), Problematic alcohol measured using the AUDIT (α = 0.65), and dissociative experiences measured using the DES-T (α = 0.90).

Group comparisons were performed using Wilcoxon rank sum tests for ordinal and continuous variables, accompanied by effect sizes calculated using Wilcoxon’s *r*. For categorical variable comparisons were made using Pearson’s Chi-square tests, and effect sizes reported using Cramer’s *V*. Both these effect size indicators range between 0 and 1, with greater values indicative of a stronger association between variables (Ferguson, 2009); in this instance grouping and outcome variables. Both are guided by similar guidelines of interpretation; where 0.2 indicates a minimally practical association, 0.5 indicates a moderate effect, and values over 0.8 are indicative of a strong effect between variables (Ferguson, 2009). All analyses applied a pairwise deletion approach, using all available data for each analysis. This method is appropriate where data are Missing Completely At Random (Allison, 2009), as was the case for data used for primary analyses in the current study (Item-level missingness: 1.32%, Little’s MCAR Test: χ2 (7228, *N* = 562) = 6408, *p* >.999).

## Results

### Case-Control Grouping

**Fig. 1:**
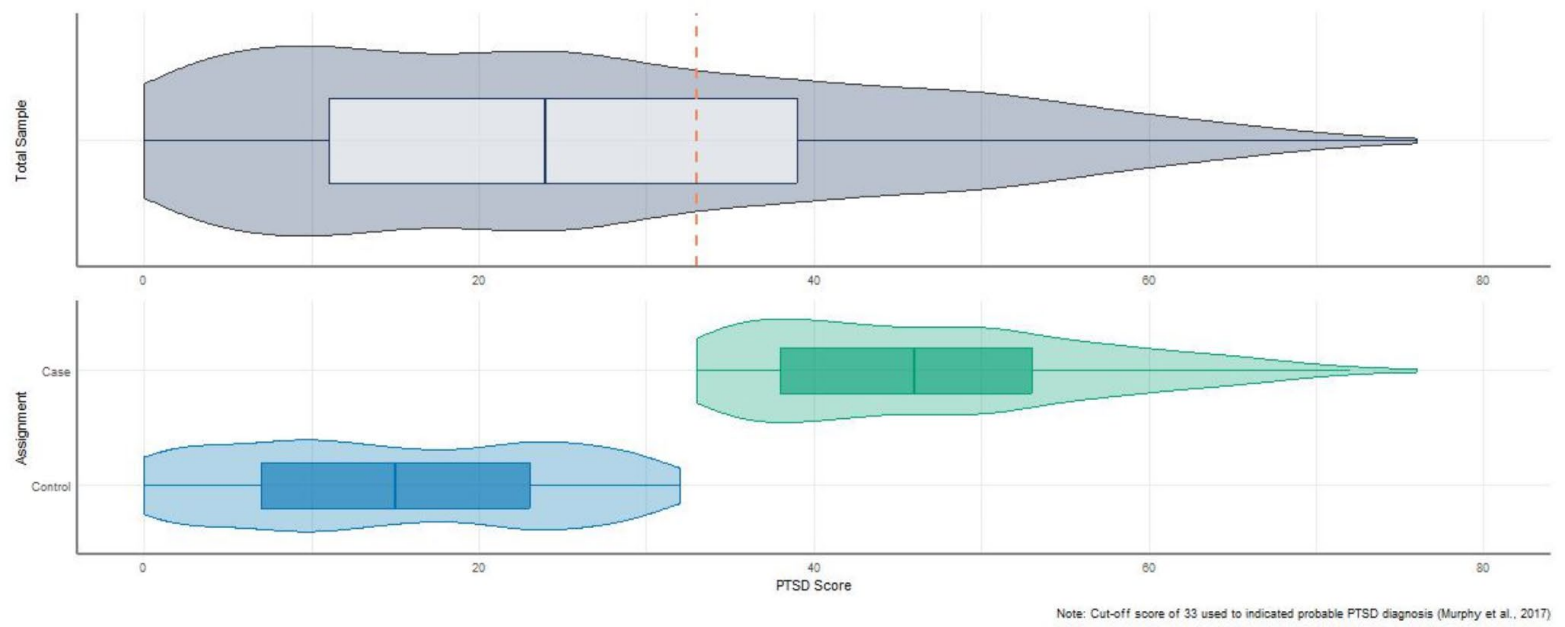
Distribution of PTSD symptom scores and case-control grouping

In total *N* = 562 participants were recruited to the study at baseline. In accordance with the study design participants were stratified into Case (*n* = 196; 34.88%) and Control (*n* = 366; 65.12%) groupings based on a cut-off score indicating *probable PTSD* using the PCL-5 (Murphy et al., 2017). The distribution of PTSD symptom ratings for the total sample, and Case/Control groupings is shown in Fig. 1. The mean PTSD symptom score for the total sample was 26.09 (*SD* = 17.95). In the Control group the mean symptom endorsement score was 15.06 (*SD* = 9.51), and in the Case group was 46.82 (*SD* = 9.92).

### Sociodemographic Summary

Demographic data collected at baseline is summarised in Table 1. Most of this sample identified as female (69.88%), heterosexual (81.27%), and frequently endorsed being married or partnered (44.92%), and Catholic (50.00%). The mean age of the sample was 43.89 (*SD* = 14.03). Statistical comparisons (see Supplementary File 1) revealed no statistically significant differences between the Case and Control groups on any sociodemographic variables with the exception of age, where those in the Case group were more likely to be older relative to the Control group (Δ*M* = + 3.21, Wilcoxon *W* = 31087.00, *p* = .011). It should be noted however that the magnitude of this effect size was small, and below the recommended minimally relevant practical threshold (Wilcoxon *r* = .108).

**Table 1:** Demographic summary of sample data stratified by case and control grouping

	Overall, *N* = 562	Control, *n* = 366	Case, *n* = 196
Gender			
Male	164 (29.23%)	105 (28.77%)	59 (30.10%)
Female	392 (69.88%)	257 (70.41%)	135 (68.88%)
Other Gender Identity	5 (0.89%)	3 (0.82%)	2 (1.02%)
Sexuality			
Heterosexual	447 (81.27%)	284 (80.00%)	163 (83.59%)
Homosexual	9 (1.64%)	6 (1.69%)	3 (1.54%)
Bisexual	11 (2.00%)	5 (1.41%)	6 (3.08%)
Other	8 (1.45%)	4 (1.13%)	4 (2.05%)
I prefer not to answer	75 (13.64%)	56 (15.77%)	19 (9.74%)
Marital Status			
Single	208 (37.08%)	140 (38.36%)	68 (34.69%)
Married or partnered	252 (44.92%)	164 (44.93%)	88 (44.90%)
Separated or divorced	57 (10.16%)	37 (10.14%)	20 (10.20%)
Widowed	35 (6.24%)	18 (4.93%)	17 (8.67%)
Other	4 (0.71%)	3 (0.82%)	1 (0.51%)
I prefer not to say	5 (0.89%)	3 (0.82%)	2 (1.02%)
Age	43.89 (14.03)	42.77 (14.10)	45.98 (13.70)
Ethnicity			
Hispano	143 (34.13%)	88 (32.96%)	55 (36.18%)
Indigena	86 (20.53%)	51 (19.10%)	35 (23.03%)
Other	6 (1.43%)	4 (1.50%)	2 (1.32%)
Do not identify with an ethnic group	133 (31.74%)	87 (32.58%)	46 (30.26%)
I prefer not to answer	51 (12.17%)	37 (13.86%)	14 (9.21%)
Religion			
Catholic	279 (50.00%)	194 (53.30%)	85 (43.81%)
Christian or Evangelical	200 (35.84%)	120 (32.97%)	80 (41.24%)
Other	22 (3.94%)	14 (3.85%)	8 (4.12%)
None	57 (10.22%)	36 (9.89%)	21 (10.82%)
Highest Level of Education Attainment			
Preschool	9 (1.61%)	6 (1.65%)	3 (1.54%)
Primary	150 (26.88%)	95 (26.17%)	55 (28.21%)
Baccalaureate (Further Education Diploma)	223 (39.96%)	146 (40.22%)	77 (39.49%)
Higher Education	22 (3.94%)	15 (4.13%)	7 (3.59%)
Professional Technical Level	30 (5.38%)	22 (6.06%)	8 (4.10%)
Technical	77 (13.80%)	50 (13.77%)	27 (13.85%)
University undergraduate	29 (5.20%)	21 (5.79%)	8 (4.10%)
None	14 (2.51%)	6 (1.65%)	8 (4.10%)
Other	4 (0.72%)	2 (0.55%)	2 (1.03%)
Economic Status			
Stratum 1–2 (Very Low - Low)	546 (98.20%)	358 (99.17%)	188 (96.41%)
Stratum 3–4 (Medium Low - Medium)	10 (1.80%)	3 (0.83%)	7 (3.59%)
Area of Residence			
Urban	391 (70.07%)	259 (71.55%)	132 (67.35%)
Rural	167 (29.93%)	103 (28.45%)	64 (32.65%)
Victim Registry			
Yes, I am registered	483 (86.40%)	319 (87.64%)	164 (84.10%)
I am not registered but I am a victim	53 (9.48%)	34 (9.34%)	19 (9.74%)
I am not registered because I am not a victim	23 (4.11%)	11 (3.02%)	12 (6.15%)
Were you or a close family member (e.g. sibling or child) forcibly recruited to be part of the revolutionary armed forces?	220 (39.64%)	132 (36.67%)	88 (45.13%)

### Endorsement of Traumatic Experiences

The most commonly endorsed potentially traumatic experience was *Forced Displacement* (88.61%), which remained true for endorsement of worst trauma experience (43.31%). The next most common trauma endorsements were *Exposure to Severe Human Suffering* (53.91%), *Combat Exposure* (53.02%), and *Physical Assault* (51.78%). Participants overall reported exposure to 8.23 potentially traumatic events on average. When compared using a Wilcox Rank Sum test it was found those in the Case group reported significantly more trauma exposures relative to the Control group (Δ*M* = + 2.88, Wilcoxon *r* = .323, *p* < .001). Those in the Case group were more likely to report exposure to potentially traumatic stressors under investigation relative to the Control group, with the exceptions of: exposure to *Natural Disaster*, *Traffic Accident*, *Toxic Substances*, and *Forced Displacement*. Further detail on the rates of endorsement and group comparisons is provided in Table 2a and b.

**Table 2a:** Lifetime trauma endorsement stratified by case and control grouping

	Overall, *N* = 562	Control, *n* = 366	Case, *n* = 196	Test Statistic	*p*-value	Effect Size
Total Trauma^1^	8.23 (4.19)	7.22 (3.85)	10.10 (4.17)	21852.500	< 0.001	0.323
Natural Disaster^*2*^	207 (36.83%)	128 (34.97%)	79 (40.31%)	1.340	0.200	0.049
Fire or Explosion^*2*^	199 (35.41%)	117 (31.97%)	82 (41.84%)	5.013	0.025	0.094
Traffic Accident^*2*^	196 (34.88%)	119 (32.51%)	77 (39.29%)	2.288	0.13	0.064
Serious Accident^*2*^	221 (39.32%)	130 (35.52%)	91 (46.43%)	5.918	0.015	0.103
Exposure to Toxic Substances^*2*^	96 (17.08%)	62 (16.94%)	34 (17.35%)	0.000	> 0.900	0.000
Physical Assault^*2*^	291 (51.78%)	174 (47.54%)	117 (59.69%)	7.072	0.008	0.112
Armed Assault^*2*^	249 (44.31%)	142 (38.80%)	107 (54.59%)	12.272	< 0.001	0.148
Sexual Assault^*2*^	97 (17.26%)	53 (14.48%)	44 (22.45%)	5.131	0.024	0.096
Unwanted Sexual Contact^*2*^	74 (13.17%)	39 (10.66%)	35 (17.86%)	5.177	0.023	0.096
Combat^*2*^	298 (53.02%)	180 (49.18%)	118 (60.20%)	5.793	0.016	0.102
Captivity^*2*^				12.644	0.002	0.150
Happened to me	172 (30.60%)	108 (29.51%)	64 (32.65%)			
Happened to a family member	67 (11.92%)	32 (8.74%)	35 (17.86%)			
Life Threatening Illness or Injury^*2*^	137 (24.38%)	62 (16.94%)	75 (38.27%)	30.343	< 0.001	0.232
Severe Human Suffering^*2*^	303 (53.91%)	167 (45.63%)	136 (69.39%)	28.052	< 0.001	0.223
Sudden Violent Death^*2*^	236 (41.99%)	119 (32.51%)	117 (59.69%)	37.605	< 0.001	0.259
Sudden Accidental Death^*2*^	143 (25.44%)	73 (19.95%)	70 (35.71%)	15.910	< 0.001	0.168
Serious Injury, Harm, or Death Caused to Another^*2*^	21 (3.74%)	6 (1.64%)	15 (7.65%)	11.216	< 0.001	0.141
Parent or Partner Ridicule^*2*^	202 (35.94%)	103 (28.14%)	99 (50.51%)	26.775	< 0.001	0.218
Physical Torture^*2*^	101 (17.97%)	43 (11.75%)	58 (29.59%)	26.370	< 0.001	0.217
Psychological Torture^*2*^	253 (45.02%)	137 (37.43%)	116 (59.18%)	23.529	< 0.001	0.205
House or Property Damaged^*2*^	188 (33.45%)	111 (30.33%)	77 (39.29%)	4.207	0.040	0.087
Forced Displacement^*2*^	498 (88.61%)	328 (89.62%)	170 (86.73%)	0.785	0.400	0.037
Other Stressful Event^*2*^	203 (36.12%)	103 (28.14%)	100 (51.02%)	27.972	< 0.001	0.223

^*1*^ Wilcoxon rank sum test; Wilcoxon *r*

^*2*^ Pearson’s Chi-squared test; Cramer’s *V*

*Note: n* (Valid Percent), *Endorsements not mutually exclusive*

**Table 2b:** Index trauma endorsement stratified by case and control grouping

	Overall, *N* = 562	Control, *n* = 366	Case, *n* = 196
**Index Trauma**			
Natural disaster	18 (3.35%)	15 (4.35%)	3 (1.55%)
Fire or Explosion	58 (10.78%)	45 (13.04%)	13 (6.74%)
Traffic accident	14 (2.60%)	6 (1.74%)	8 (4.15%)
Accident at work, at home or during recreational activities	8 (1.49%)	2 (0.58%)	6 (3.11%)
Exposure to toxic substances	2 (0.37%)	2 (0.58%)	0 (0.00%)
Physical assault	13 (2.42%)	10 (2.90%)	3 (1.55%)
Armed assault	17 (3.16%)	6 (1.74%)	11 (5.70%)
Sexual assault	20 (3.72%)	9 (2.61%)	11 (5.70%)
Other unwanted sexual activity	5 (0.93%)	3 (0.87%)	2 (1.04%)
Combat or exposure to a war zone	29 (5.39%)	18 (5.22%)	11 (5.70%)
Captivity	12 (2.23%)	7 (2.03%)	5 (2.59%)
Life-threatening illness or injury	4 (0.74%)	3 (0.87%)	1 (0.52%)
Severe human suffering	10 (1.86%)	4 (1.16%)	6 (3.11%)
Violent unexpected death	57 (10.59%)	30 (8.70%)	27 (13.99%)
Unexpected accidental death	7 (1.30%)	5 (1.45%)	2 (1.04%)
Serious injury, harm, or death caused to someone else	0 (0.00%)	0 (0.00%)	0 (0.00%)
Parent/partner verbal abuse	10 (1.86%)	5 (1.45%)	5 (2.59%)
Physical torture	2 (0.37%)	1 (0.29%)	1 (0.52%)
Psychological torture	12 (2.23%)	6 (1.74%)	6 (3.11%)
Home or property damaged	1 (0.19%)	1 (0.29%)	0 (0.00%)
Forced displacement	233 (43.31%)	163 (47.25%)	70 (36.27%)
Another stressful experience or event	6 (1.12%)	4 (1.16%)	2 (1.04%)

Note: *n* (Valid Percent)

### Mental Health Outcomes

The reported rate of probable diagnoses for mental ill-health outcomes assessed in the current study are reported in Table 3. A score of 10 or greater was used as this is a well-established cut-off indicative of probable depression and anxiety using the PHQ-9 and GAD-7 respectively (Kroenke et al., 2001; Spitzer et al., 2006). A cut off score of 10 or greater was similarly applied to screen for potential problematic alcohol use (Babor et al., 2001).

**Table 3:** Mental health outcome comparison between case and control groups

	Overall, *N* = 562	Control, *n* = 366	Case, *n* = 196	Test Statistic	*p*-value	Effect Size
Total Depression Score^*1*^	6.96 (6.67)	4.12 (4.84)	12.01 (6.77)	10233.000	< 0.001	0.566
Probable Depression ^*2*^	157 (29.90%)	43 (12.80%)	114 (60.32%)	128.000	< 0.001	0.494
Total Anxiety Score^*1*^	6.60 (5.89)	4.13 (4.48)	11.15 (5.45)	10078.500	< 0.001	0.576
Probable Anxiety ^*2*^	148 (27.56%)	44 (12.64%)	104 (55.03%)	108.088	< 0.001	0.449
Probable Alcohol Use Disorder ^*2*^	22 (13.66%)	11 (9.82%)	11 (22.45%)	3.599	0.058	0.150
Number of Probable Common Mental Health Diagnoses ^*1*^	0.93 (1.16)	0.27 (0.59)	2.17 (0.92)	3668.000	< 0.001	0.806
Dissociative Experiences^*1*^	1.07 (1.84)	0.88 (1.70)	1.43 (2.02)	26942.500	< 0.001	0.179

^*1*^ Wilcoxon rank sum test; Wilcoxon *r*

^*2*^ Pearson’s Chi-squared test; Cramer’s *V*

Note:*n* (Valid Percent)

Differences between groups were assessed using Wilcoxon Rank Sum for continuous variables, and Chi-square test for categorical variable comparisons. Consistent with expectations a statistically significant difference was observed between groups for the majority of mental health outcomes under investigation, with the exception of problematic alcohol use (*p* = .058). Additionally, the Case group reported significantly greater rates of comorbidity (cooccurring PTSD, Anxiety, Depression, or Alcohol Misuse) relative to the Control group (Δ*M* = + 1.90, Wilcoxon *r* = .806, *p* < .001). Almost half of the study sample (*n* = 268, 47.69%) screened positively for at least one of these probable mental ill-health outcomes (see Supplemental File 1).

## Discussion

The current study provides a detailed summary of the design and methods of the psychosocial arm of the MI-VIDA research programme, detailing the study rationale and the technical aspects of the data collection procedure, including instruments used in the wider study.

Approximately one third of this trauma exposed sample screened positive for probable PTSD. This rate is notably higher than population prevalence estimates of PTSD morbidity (Gaviria et al., 2016; Koenen et al., 2017), however is in line with review evidence suggesting approximately one third of those exposed to potentially traumatic events report pathological distress (Galatzer-Levy et al., 2018), and with expectations for those exposed to multiple traumatic stressors to be significantly more likely to experience PTSD (Breslau et al., 2008). Indeed, previous evidence from Colombia has demonstrated that those in regions exposed to conflict are at increased risk for PTSD pathology (Tamayo-Agudelo & Bell, 2019), as are those in areas with greater poverty and less educational attainment (Botero-Rodríguez et al., 2021). These findings are therefore consistent with expectations given the study design and sampling strategy.

This current study also reports a significant burden of common mental health disorders in the cohort with almost half (47.69%) screening positive for a probable diagnosis of one or more psychopathological disorders. This endorsement rate is notably elevated in comparison to previous estimates of psychological morbidity in the Colombian general population (Gomez-Restrepo et al., 2016; Koenen et al., 2017). It should be noted however that the current study sample was comprised specifically of trauma-exposed individuals residing in areas historically effected by conflict and internal displacement (see Jiménez Ortega et al., 2019). Extant evidence suggests that internally displaced persons are at significantly elevated risk for common mental health disorders and PTSD, with similar rates of PTSD and depression observed in these populations in different contexts (Roberts et al., 2008; Rofo et al., 2023); hence this finding is broadly consistent with expectations.

Further to this, those in the Control group reported significantly fewer trauma experiences and mental health morbidities. Notably rates of probable generalized anxiety, major depression, and dissociative experience were each elevated in the Case group. This is in keeping with previous research that has suggested that trauma histories and residence in post-conflict regions are associated with increased risk for these common mental health disorders (see Charlson et al., 2016; Jeffery, 2023). PTSD is likewise understood to be a highly comorbid condition (Brady et al., 2000; Galatzer-Levy et al., 2013) and as such it is anticipated that the Case group would report significantly greater psychopathology symptoms.

Notably however, a non-significant difference was observed between groups regarding probable alcohol use disorder. Alcohol use disorders have been previously noted to be prevalent in Latin America, with an estimated prevalence rate of approximately 9% in the Colombian population (Rincon-Hoyos et al., 2016). Alcohol use disorders have however been more associated with males compared to females in the region (Rincon-Hoyos et al., 2016). The nonsignificant results pertaining to alcohol use disorder in the current study may therefore be owing to the greater number of female participants in this study.

There were minimal differences between the Case and Control groups regarding sociodemographic variables, with the exception that those in the Case group were slightly older than the Control group. This runs contrary to expectations given that older age is often considered a protective factor for posttraumatic stress reactions (Brewin et al., 2000), however it may be considered that those older in age are more likely to have experienced traumatic stressors associated with the armed conflict and for a longer period of time (see Jiménez Ortega et al., 2019). Older people may have accumulated more traumatic events over times of high violence during the decades of conflict with FARC guerrillas since its beginnings in the 1960s until the end of 2012, when the peace talks started and the violence began to decrease significantly (Casas, 2021). It should be noted though that this effect size was found to be below the minimally relevant practical threshold, and as such extrapolating implications is cautioned. The demographic similarity between these groups may be considered a relative strength of these data and allow for valuable contribution and robust interpretations in this broader investigation of risk and resilience factors for traumatic stress.

The findings reported in the current study chiefly relate to the stated goal of describing the participant sample and Case-Control methodology adopted by the MI-VIDA investigation, however these initial descriptive findings provide cursory implications for further investigations. These findings highlight: probable PTSD as a concern for approximately one third of this trauma-exposed sample, and potential correlates with this outcome that call for additional investigation into factors which may convey risk or resilience for traumatic stress outcomes. The current study offers a methodological overview of the MI-VIDA study and assessment limited to the core data and features within. The broader investigation seeks to conduct more elaborate analysis and in turn provide a greater depth of understanding of the context of risk and resilience factors for posttraumatic outcomes in the Colombian context. This greater depth of understanding will facilitate the development of mental health policy, services and treatments in the region as aligned to the goals of the PDET programme.

### Strength and Limitations

This study possesses unique strengths and novelty in assessment of PTSD risk and resilience in a trauma-exposed Latin American sample. Despite this it should be acknowledged that due to the purposeful sampling of those endorsing trauma exposure in areas historically affected by conflict the study population may not be representative of the wider Colombian population in terms of demographics, trauma experiences, and mental health difficulties. Generalisation of these results should thus be cautioned. The sample within the current study may, as intended, be aptly used for Case-Control comparisons. Indeed, the distribution of Case and Control group assignment is broadly in line with prevalence estimates suggesting up to one third of trauma exposed samples experiencing probable PTSD (Santiago et al., 2013). While unbalance is acknowledged between the Case and Control groups this should not be considered to invalidate results (see Cheng et al., 2014).

The current investigation addresses shortcomings of previous studies through longitudinal collection of comprehensive data related to risk and resiliency factors for PTSD. Measures used however should be acknowledged to be self-report in nature and hence potentially limited by satisficing and recall biases.

### Research Agenda and Future Directions

This programme of research seeks to evaluate biopsychosocial factors associated with the development of PTSD in this Colombian sample. These data will be used to assess a myriad of potential risk and resiliency factors highlighted by previous research. Using this longitudinal case-control design we hope to identify factors that contribute to the development of psychopathology over time. The current study seeks to provide an overview of the methodology (and measures used within) of the MI-VIDA investigation.

While several appropriate measures exist for the assessment of psychological morbidity, risk, and resiliency factors it is noted that some necessitated translation for the current study. For this reason, exploration of the psychometric properties of these measures is warranted to ensure their validity.

Finally, while beyond the scope of this investigation the extant literature would benefit from up-to-date evidence on the incidence of traumatic stress and mental ill-health in Colombia. This investigation sought to identify a cohort of Colombian residents exposed to trauma, however as there is a dearth of current psychological population-level evidence the generalisation of these research findings cannot be guaranteed. Future research should consider the value in undertaking more epidemiological work investigating psychological morbidity more broadly in this context.

## Conclusion

The MI-VIDA study seeks to evaluate evidenced risk and resiliency factors on the development of PTSD and other psychopathologies in a sample of trauma-exposed Colombian residents. This reporting presents the methodology and initial findings reflecting recruitment of a trauma-exposed and under-researched group in the Global South. The findings of this investigation stand to make a significant contribution to current understanding of post-trauma psychological risk and resilience in Latin America. The employment of a longitudinal design additionally promises valuable insight into trajectories and development of psychopathology in this group.

## Electronic Supplementary Material

Below is the link to the electronic supplementary material.

Supplementary Material 1

Supplementary Material 2

## Data Availability

Participants did not provide consent for their data to be made publicly available at the time of collection. Anonymised data supporting findings of this study will be made available from the corresponding author on reasonable request.

## Code availability

Analyses and figures produced using R Studio (R version 4.4.1, R Studio Build 764). Code for this study are made available as an R Markdown document in Supplementary File 1.
