# Supplementary material for: Estudio De La Vida Bajo Estres: Methodological Overview and Baseline Data Analysis of a Case-Control Investigation of Risk and Resiliency Factors for Traumatic Stress in Colombia
Source: J Psychopathol Behav Assess. 2025 Mar 1;47(1):25. doi: 10.1007/s10862-025-10203-1 (PMC11872984; doi:10.1007/s10862-025-10203-1)
Supplement: Supplementary file 1 — Supplementary Material 1 [file 10862_2025_10203_MOESM1_ESM.docx]

**Supplemental Table 1**: Survey Measure Summary Details

| Measure Name | Time  point(s) | English  Reference | Spanish Version | Spanish Reference |
| --- | --- | --- | --- | --- |
| Demographics | T1 | - | N |  |
| Victims Official Registry | T1, T3 | - | N |  |
| Psychosocial Functioning during COVID-19 Questionnaire | T1, T3, Abridged version (7-items) at T2 | Arnold, T., Kunicki, Z. J., Rogers, B. G., Haubrick, K. K., Klasko-Foster, L., Norris, A. L., . . . Cohen, S. A. (2021). Validating the Psychosocial Functioning during COVID-19 Questionnaire among a Sample of Informal Caregivers. Gerontol Geriatr Med, 7, 2333721421997200. doi: 10.1177/2333721421997200 | N |  |
| Stressful Life Events | T1, T3 | Weathers, F. W., Blake, D.D., Schnurr, P.P., Kaloupek, D.G., Marx, B.P., Keane, T.M. (2013). The Life Events Checklist for DSM-5 (LEC-5). Retrieved from www.ptsd.va.gov; Taylor, L. K. (2016). Impact of political violence, social trust, and depression on civic participation in Colombia. Peace and Conflict: Journal of Peace Psychology, 22(2), 145-152. doi: 10.1037/pac0000139 | N |  |
| Adverse Childhood Experiences Scale | T1 | Felitti, V. J., Anda, R. F., Nordenberg, D., Williamson, D. F., Spitz, A. M., Edwards, V., . . . Marks, J. S. (1998). Relationship of Childhood Abuse and Household Dysfunction to Many of the Leading Causes of Death in Adults. American Journal of Preventive Medicine, 14(4), 245-258. doi: 10.1016/s0749-3797(98)00017-8 | Y | Von Sneidern Calero, E. (2016). Asociación del índice de experiencias adversas de la infancia (ACE) con el desarrollo infantil en niños (as) preescolares de Barú, Bolívar (Maestría en Salud Pública Masters). Universidad de los Andes, Retrieved from http://hdl.handle.net/1992/13313 |
| Community Anti-social Behaviour | T1 | Taylor, L. K. (2016). Impact of political violence, social trust, and depression on civic participation in Colombia. Peace and Conflict: Journal of Peace Psychology, 22(2), 145-152. doi: 10.1037/pac0000139 | Y | Taylor, L. K. (2016). Impact of political violence, social trust, and depression on civic participation in Colombia. Peace and Conflict: Journal of Peace Psychology, 22(2), 145-152. doi: 10.1037/pac0000139 |
| Help Seeking from NIVHWS | T1 | Armour, C., McGlinchey, E., Ross, J. (2021). The Health and Wellbeing of Armed Forces Veterans in Northern Ireland: The Results of a Cross-sectional Psychological Wellbeing Survey. Retrieved from http://niveteranstudy.org/mental-health | N |  |
| Dimensions of Anger Reactivity - Revised | T1 | Kannis-Dymand, L., Salguero, J. M., Ramos-Cejudo, J., & Novaco, R. W. (2019). Dimensions of Anger Reactions-Revised (DAR-R): Validation of a brief anger measure in Australia and Spain. J Clin Psychol, 75(7), 1233-1248. doi: 10.1002/jclp.22757 | Y | Kannis-Dymand, L., Salguero, J. M., Ramos-Cejudo, J., & Novaco, R. W. (2019). Dimensions of Anger Reactions-Revised (DAR-R): Validation of a brief anger measure in Australia and Spain. J Clin Psychol, 75(7), 1233-1248. doi: 10.1002/jclp.22757 |
| Brief Cope Scale | T2 | Carver, C. S. (1997). You want to measure coping but your protocol's too long: consider the brief COPE. Int J Behav Med, 4(1), 92-100. doi: 10.1207/s15327558ijbm0401_6 | Y | Perczek, R., Carver, C. S., Price, A. A., & Pozo-Kaderman, C. (2000). Coping, mood, and aspects of personality in Spanish translation and evidence of convergence with English versions. J Pers Assess, 74(1), 63-87. doi: 10.1207/S15327752JPA740105 |
| Connor-Davidson Resilience Scale | All | Connor, K. M., & Davidson, J. R. (2003). Development of a new resilience scale: the Connor-Davidson Resilience Scale (CD-RISC). Depress Anxiety, 18(2), 76-82. doi: 10.1002/da.10113 | Y | Connor, K. M., & Davidson, J. R. (2003). Development of a new resilience scale: the Connor-Davidson Resilience Scale (CD-RISC). Depress Anxiety, 18(2), 76-82. doi: 10.1002/da.10113 |
| Trauma Appraisal Questionnaire | All | DePrince, A. P., Zurbriggen, E. L., Chu, A. T., & Smart, L. (2010). Development of the Trauma Appraisal Questionnaire. Journal of Aggression, Maltreatment & Trauma, 19(3), 275-299. doi: 10.1080/10926771003705072 | N |  |
| Meaning in Life Questionnaire | T1 | Steger, M. F., Frazier, P., Oishi, S., & Kaler, M. (2006). The meaning in life questionnaire: Assessing the presence of and search for meaning in life. Journal of Counseling Psychology, 53(1), 80-93. doi: 10.1037/0022-0167.53.1.80 | Y | Steger, M. F., & Zaccagnini, J. L. The Meaning in Life Questionnaire (Spanish verison). Retrieved from http://www.michaelfsteger.com/ |
| The 10-item persecution and deservedness scale | T1 | Melo, S., Corcoran, R., Shryane, N., & Bentall, R. P. (2009). The persecution and deservedness scale. Psychol Psychother, 82(Pt 3), 247-260. doi: 10.1348/147608308X398337 | Y | Valiente, C., Contreras, A., Trucharte, A., Peinado, V., & Espinosa, R. (2020). Psychometric properties and normative data of the Spanish version of short form persecution and deservedness scale. Psychosis, 13(2), 130-142. doi: 10.1080/17522439.2020.1834605 |
| State Optimism Measure - 7 | T1 | Millstein, R. A., Chung, W. J., Hoeppner, B. B., Boehm, J. K., Legler, S. R., Mastromauro, C. A., & Huffman, J. C. (2019). Development of the State Optimism Measure. Gen Hosp Psychiatry, 58, 83-93. doi: 10.1016/j.genhosppsych.2019.04.002 | N |  |
| Single Item Happiness Scale | All | Abdel-Khalek, A. M. (2006). Measuring Happiness with a Single-Item Scale. Social Behavior and Personality: an international journal, 34(2), 139-150. doi: 10.2224/sbp.2006.34.2.139 | N |  |
| UCLA Loneliness Scale | T2 | Hughes, M. E., Waite, L. J., Hawkley, L. C., & Cacioppo, J. T. (2004). A Short Scale for Measuring Loneliness in Large Surveys: Results From Two Population-Based Studies. Res Aging, 26(6), 655-672. doi: 10.1177/0164027504268574 | N |  |
| The Warwick-Edinburgh Mental Wellbeing Scale | T1 | Tennant, R., Hiller, L., Fishwick, R., Platt, S., Joseph, S., Weich, S., . . . Stewart-Brown, S. (2007). The Warwick-Edinburgh Mental Well-being Scale (WEMWBS): development and UK validation. Health Qual Life Outcomes, 5, 63. doi: 10.1186/1477-7525-5-63 | Y | Lopez, M. A., Gabilondo, A., Codony, M., Garcia-Forero, C., Vilagut, G., Castellvi, P., . . . Alonso, J. (2013). Adaptation into Spanish of the Warwick-Edinburgh Mental Well-being Scale (WEMWBS) and preliminary validation in a student sample. Qual Life Res, 22(5), 1099-1104. doi: 10.1007/s11136-012-0238-z |
| Difficulties in Emotional Regulation Scale Short Form (DERS-SF) |  | Kaufman, E. A., Xia, M., Fosco, G., Yaptangco, M., Skidmore, C. R., & Crowell, S. E. (2015). The Difficulties in Emotion Regulation Scale Short Form (DERS-SF): Validation and Replication in Adolescent and Adult Samples. Journal of Psychopathology and Behavioral Assessment, 38(3), 443-455. doi: 10.1007/s10862-015-9529-3 | Y | University of Miami. (2016). Difficulties in Emotion Regulation Scale Short form (Spanish Translation). Retrieved from https://elcentro.sonhs.miami.edu/research/measures-library/ders-sf/index.html |
| Context Sensitivity Index | T1 | Bonanno, G. A., Maccallum, F., Malgaroli, M., & Hou, W. K. (2020). The Context Sensitivity Index (CSI): Measuring the Ability to Identify the Presence and Absence of Stressor Context Cues. Assessment, 27(2), 261-273. doi: 10.1177/1073191118820131 | N |  |
| Coping Flexibility Scale-Revised | T2 | Kato, T. (2020). Examination of the Coping Flexibility Hypothesis Using the Coping Flexibility Scale-Revised. Front Psychol, 11, 561731. doi: 10.3389/fpsyg.2020.561731 | N |  |
| Cognitive Flexibility Scale | T1 | Martin, M. M., & Rubin, R. B. (2016). A New Measure of Cognitive Flexibility. Psychological Reports, 76(2), 623-626. doi: 10.2466/pr0.1995.76.2.623 | N |  |
| Multidimensional Scale of Perceived Social Support | All | Zimet, G. D., Dahlem, N. W., Zimet, S. G., & Farley, G. K. (1988). The Multidimensional Scale of Perceived Social Support. Journal of Personality Assessment, 52(1), 30-41. doi: 10.1207/s15327752jpa5201_2 | Y | Cobb, C. L., & Xie, D. (2015). Structure of the Multidimensional Scale of Perceived Social Support for Undocumented Hispanic Immigrants. Hispanic Journal of Behavioral Sciences, 37(2), 274-281. doi: 10.1177/0739986315577894 |
| World Values Survey | T1 | World Values Survey (2018). Colombia (2018). World Values Survey Wave 7 (2017-2020). Retrieved from https://www.worldvaluessurvey.org/WVSDocumentationWV7.jsp | Y | World Values Survey (2018). Colombia (2018). World Values Survey Wave 7 (2017-2020). Retrieved from https://www.worldvaluessurvey.org/WVSDocumentationWV7.jsp |
| Social and Personal Identities Scale | T1 | Nario-Redmond, M. R., Biernat, M., Eidelman, S., & Palenske, D. J. (2012). The Social and Personal Identities Scale: A Measure of the Differential Importance Ascribed to Social and Personal Self-Categorizations. Self and Identity, 3(2), 143-175. doi: 10.1080/13576500342000103 | N |  |
| Marlowe-Crowne Social Desirability – Short Form | T1 | Reynolds, W. M. (1982). Development of reliable and valid short forms of the marlowe-crowne social desirability scale. Journal of Clinical Psychology, 38(1), 119-125. doi: 10.1002/1097-4679(198201)38:1<119::Aid-jclp2270380118>3.0.Co;2-i | Y | Cosentino, A. C., & Castro Solano, A. (2008). Adaptación y validación Argentina de la marlowe-crowne socialdesirability scale. [Argentine adaptation and validation of the marlowe-crowne social desirability scale]. Interdisciplinaria, 25(2), 197-216. |
| Eysenck Lie Scale (EPQR-S) | All | Eysenck, H. J., & Eysenck, S. B. G. (1992). Manual for the Eysenck Personality Questionnaire Revised. San Diego, CA: Educational and Industrial Testing Service. | N |  |
| PTSD Checklist for DSM-5 | All | Weathers, F. W., Litz, B.T., Keane, T.M., Palmieri, P.A., Marx, B.P., Schnurr, P.P. (2013). The PTSD Checklist for DSM-5 (PCL-5). Retrieved from www.ptsd.va.gov | Y | Rendon, M. J. (2015). The Cultural Adaptation of the Clinician-Administered PTSD Scale for Spanish-Speaking Latinos with Limited English Proficiency in the United States. (Doctor of Philosophy (PhD) PhD). University of Miami, Retrieved from https://scholarship.miami.edu/discovery/delivery?vid=01UOML_INST:ResearchRepository&repId=12355317860002976 |
| International Trauma Questionnaire | All | Cloitre, M., Shevlin, M., Brewin, C. R., Bisson, J. I., Roberts, N. P., Maercker, A., . . . Hyland, P. (2018). The International Trauma Questionnaire: development of a self-report measure of ICD-11 PTSD and complex PTSD. Acta Psychiatr Scand, 138(6), 536-546. doi: 10.1111/acps.12956 | Y | Carmen Fernández-Fillol, J. C. D., Natalia Hidalgo-Ruzzante, Miguel Pérez-García. (2018). Cuestionario Internacional Sobre Estrés Postraumático (ITQ). Retrieved from https://www.traumameasuresglobal.com/itq |
| Post Traumatic Growth Inventory – Short Form | All | Cann, A., Calhoun, L. G., Tedeschi, R. G., Taku, K., Vishnevsky, T., Triplett, K. N., & Danhauer, S. C. (2010). A short form of the Posttraumatic Growth Inventory. Anxiety Stress Coping, 23(2), 127-137. doi: 10.1080/10615800903094273 | Y | Castro, M. C., Delgado, J. B., Alvarado, E. R., & Rovira, D. P. (2015). Spanish Adaptation and Validation of the Posttraumatic Growth Inventory-Short Form. Violence Vict, 30(5), 756-769. doi: 10.1891/0886-6708.VV-D-13-00165 |
| Patient Health Questionnaire | All | Kroenke, K., Spitzer, R. L., & Williams, J. B. (2001). The PHQ-9: validity of a brief depression severity measure. J Gen Intern Med, 16(9), 606-613. doi: 10.1046/j.1525-1497.2001.016009606.x | Y | Miranda, C. A. C., & Scoppetta, O. (2018). Factorial structure of the Patient Health Questionnaire-9 as a depression screening instrument for university students in Cartagena, Colombia. Psychiatry Res, 269, 425-429. doi: 10.1016/j.psychres.2018.08.071 |
| Generalised Anxiety Disorder Scale | All | Spitzer, R. L., Kroenke, K., Williams, J. B., & Lowe, B. (2006). A brief measure for assessing generalized anxiety disorder: the GAD-7. Arch Intern Med, 166(10), 1092-1097. doi: 10.1001/archinte.166.10.1092 | Y | García-Campayo, J., Zamorano, E., Ruiz, M.A. et al. Cultural adaptation into Spanish of the generalized anxiety disorder-7 (GAD-7) scale as a screening tool. Health Qual Life Outcomes 8, 8 (2010). https://doi.org/10.1186/1477-7525-8-8 |
| Dissociative Experiences Scale Taxon | All | Waller, N., Putnam, F. W., & Carlson, E. B. (1996). Types of dissociation and dissociative types: A taxometric analysis of dissociative experiences. Psychological Methods, 1(3), 300-321. doi: 10.1037/1082-989x.1.3.300 | Y | Martinez, A. (1995). The use of the Dissociative Experiences Scale in Puerto Rico. Dissociation, 3(1), 14-23. ; Icarán, E., Colom, R., Orengo-García, F. (1996). Estudio de validación de la escala de experiencias disociativas con muestra de población española. [Validation study of the dissociative experiences scale in Spanish population sample]. Actas Luso-Espanolas de Neurologia, Psiquiatria y Ciencias Afines, 24(1), 7–10. |
| Insomnia Severity Index | All | Bastien, C. (2001). Validation of the Insomnia Severity Index as an outcome measure for insomnia research. Sleep Medicine, 2(4), 297-307. doi: 10.1016/s1389-9457(00)00065-4 | Y | Fernandez-Mendoza, J., Rodriguez-Munoz, A., Vela-Bueno, A., Olavarrieta-Bernardino, S., Calhoun, S. L., Bixler, E. O., & Vgontzas, A. N. (2012). The Spanish version of the Insomnia Severity Index: a confirmatory factor analysis. Sleep Med, 13(2), 207-210. doi: 10.1016/j.sleep.2011.06.019; Recived emasure and liscence from ePROVIDE. |
| Fear of Sleep Inventory-Short Form | T1 | Pruiksma, K. E., Taylor, D. J., Ruggero, C., Boals, A., Davis, J. L., Cranston, C., . . . Zayfert, C. (2014). A psychometric study of the Fear of Sleep Inventory-Short Form (FoSI-SF). J Clin Sleep Med, 10(5), 551-558. doi: 10.5664/jcsm.3710 | N |  |
| Pittsburgh Sleep Quality Index | T2 | Carpenter, J. S., & Andrykowski, M. A. (1998). Psychometric evaluation of the pittsburgh sleep quality index. Journal of Psychosomatic Research, 45(1), 5-13. doi: 10.1016/s0022-3999(97)00298-5 | Y | Tomfohr, L. M., Schweizer, C. A., Dimsdale, J. E., & Loredo, J. S. (2013). Psychometric characteristics of the Pittsburgh Sleep Quality Index in English speaking non-Hispanic whites and English and Spanish speaking Hispanics of Mexican descent. J Clin Sleep Med, 9(1), 61-66. doi: 10.5664/jcsm.2342; Rodriguez-Morales, A. J., Ochoa-Orozco, S. A., Sánchez-Castaño, D. M., Corzo-Romero, J. D., Erazo-Landázuri, J. X., Lopez-Caicedo, D. F., . . . Lagos-Grisales, G. J. (2018). Pittsburgh Sleep Quality Index (PSQI) and Fatigue Severity Scale (FSS) assessment in patients with post-Chikungunya chronic inflammatory rheumatism: a comparative study of a 2-year follow-up cohort in La Virginia, Risaralda, Colombia. International Journal of Infectious Diseases, 73. doi: 10.1016/j.ijid.2018.04.4092 |
| Alcohol Use Disorders Identification Test | All | Babor, T. F., de la Fuente, J. R., Saunders, J., & Grant, M. (1992). The Alcohol Use Disorders Identification Test: Guidelines for use in primary health care. Geneva, Switzerland: World Health Organization | Y | Ballester, L., Alayo, I., Vilagut, G., Almenara, J., Cebria, A. I., Echeburua, E., . . . On Behalf Of The Universal Study, G. (2021). Validation of an Online Version of the Alcohol Use Disorders Identification Test (AUDIT) for Alcohol Screening in Spanish University Students. Int J Environ Res Public Health, 18(10). doi: 10.3390/ijerph18105213 |
| Single-Question Screening Test for Drug Use | All | Smith, P. C., Schmidt, S. M., Allensworth-Davies, D., & Saitz, R. (2010). A single-question screening test for drug use in primary care. Arch Intern Med, 170(13), 1155-1160. doi: 10.1001/archinternmed.2010.140 | N |  |
