# Supplementary material for: Estudio De La Vida Bajo Estres: Methodological Overview and Baseline Data Analysis of a Case-Control Investigation of Risk and Resiliency Factors for Traumatic Stress in Colombia
Source: J Psychopathol Behav Assess. 2025 Mar 1;47(1):25. doi: 10.1007/s10862-025-10203-1 (PMC11872984; doi:10.1007/s10862-025-10203-1)
Supplement: Supplementary file 2 — Supplementary Material 2 [file 10862_2025_10203_MOESM2_ESM.html]

Methodological Overview and Baseline Data Analysis of a Case-Control Investigation of Risk and Resiliency Factors for Traumatic Stress in Colombia


# Methodological Overview and Baseline Data Analysis of a Case-Control Investigation of Risk and Resiliency Factors for Traumatic Stress in Colombia

### Supplemental File 1

#### 2024-10-04

## Data and Function Loading

```
knitr::opts_chunk$set(echo = TRUE, collapse = TRUE, out.width = "100%",               # Standard Markdown chunk behaviour        
                      warning = FALSE, message = FALSE)                         # Streamline R Markdown output with removed messages
```

```
options(width = 600)
library(haven)
library(gtsummary)
library(tidyverse)
library(rstatix)
library(coin)

data <- read.csv("MIVIDA Methods Paper Data.csv")

data <- data |> dplyr::filter(!is.na(pclscore))


Eff_size_r <- function(data, variable, by, ...) {
                     rstatix::wilcox_effsize(data, as.formula(glue::glue("{variable} ~ {by}", ci = TRUE, conf.level = 0.95)))$effsize
}

Eff_size_V <- function(data, variable, by, ...) {
                       table(data[[variable]], data[[by]]) |> 
                       rstatix::cramer_v()
}

## Internal Reliability for measures in analyses
#  PCL-5
data |> dplyr::select(pcl_1:pcl_20) |> 
ltm::cronbach.alpha(na.rm = TRUE)
## 
## Cronbach's alpha for the 'dplyr::select(data, pcl_1:pcl_20)' data-set
## 
## Items: 20
## Sample units: 562
## alpha: 0.949

#  PHQ-9
data |> dplyr::select(phq1:phq9) |> 
ltm::cronbach.alpha(na.rm = TRUE)
## 
## Cronbach's alpha for the 'dplyr::select(data, phq1:phq9)' data-set
## 
## Items: 9
## Sample units: 562
## alpha: 0.91

#  GAD-7
data |> dplyr::select(gad1:gad7) |> 
ltm::cronbach.alpha(na.rm = TRUE)
## 
## Cronbach's alpha for the 'dplyr::select(data, gad1:gad7)' data-set
## 
## Items: 7
## Sample units: 562
## alpha: 0.915

#  AUDIT
data |> dplyr::select(audit1:audit10) |> 
ltm::cronbach.alpha(na.rm = TRUE)
## 
## Cronbach's alpha for the 'dplyr::select(data, audit1:audit10)' data-set
## 
## Items: 10
## Sample units: 562
## alpha: 0.654

# Dissociative Experiences
data |> dplyr::select(des1:des8) |> 
ltm::cronbach.alpha(na.rm = TRUE)
## 
## Cronbach's alpha for the 'dplyr::select(data, des1:des8)' data-set
## 
## Items: 8
## Sample units: 562
## alpha: 0.898
```

## Summary Statistics

```
table1 <-
data |> 
  mutate(Assignment      = factor(pclgroup, labels = c("Control", "Case")),
         Gender          = data$dem2.factor,
         Sexuality       = dem6.factor,
         Martial_Status  = dem4.factor,
         Age             = dem3,
         Ethnicity       = dem11.factor,
         Religion        = dem12.factor,
         Education       = dem14.factor,
         Economic_Stat   = dem17.factor,
         Living_Area     = dem8.factor,
         Victim_Registry = vic1.factor,
         FARC_Involved   = farc.factor) |> 
  # Factors with limited numbers collapsed to minimise participant identification
  mutate(Gender = recode_factor(Gender, 
                                "Man" = "Male",
                                "Female" = "Female",
                                "Transgender man" = "Other Gender Identity",
                                "Transgender woman" = "Other Gender Identity",
                                "Transgender/gender  fluid" = "Other Gender Identity",
                                "Other" = "Other Gender Identity"),
         Ethnicity = recode(Ethnicity, 
                            "Gitano" = "Other",
                            "Other - Please specify" = "Other"),
         Economic_Stat = recode_factor(Economic_Stat,
                                       "Stratum 1 (Very low)" = "Stratum 1-2 (Very Low - Low)",
                                       "Stratum 2 (Low)" = "Stratum 1-2 (Very Low - Low)",
                                       "Stratum 3 (Medium Low)" = "Stratum 3-4 (Medium Low - Medium)",
                                       "Stratum 4 (Medium)" = "Stratum 3-4 (Medium Low - Medium)"),
         Religion = recode_factor(Religion,
                                   "Catholic" = "Catholic",
                                   "Protestant" = "Other",
                                   "Jeovah Witness" = "Other",
                                   "Other  - Please Specify" = "Other"),
         Sexuality = recode_factor(Sexuality,
                                   "Heterosexual" = "Heterosexual")
         ) |> 
  select(Assignment, Gender, Sexuality, Martial_Status, Age, Ethnicity, Religion, 
         Education, Economic_Stat, Living_Area, Victim_Registry, FARC_Involved) |> 
  tbl_summary(by = Assignment,
    label = list(Economic_Stat ~ "Economic Status",
                 Living_Area   ~ "Area of Residence",
                 FARC_Involved ~ "Were you or a close family member (e.g. sibling or child) 
                                  forcibly recruited to be part of the revolutionary armed forces?"),
    type = c(Age) ~  "continuous" ,
    statistic = all_continuous() ~ "{mean} ({sd})",
    digits = list(all_continuous() ~ 2, all_categorical() ~ c(0, 2)),
    missing = "no") |> 
    add_overall()   |> 
    add_stat(fns = list(all_continuous()  ~ Eff_size_r,
                      all_categorical() ~ Eff_size_V)) |> 
  modify_header(add_stat_1 ~ "**Effect Size**"); table1
```

| **Characteristic** | **Overall**  N = 5621 | **Control**  N = 3661 | **Case**  N = 1961 | **Effect Size** |
| --- | --- | --- | --- | --- |
| Gender |  |  |  | 0.018 |
| Male | 164 (29.23%) | 105 (28.77%) | 59 (30.10%) |  |
| Female | 392 (69.88%) | 257 (70.41%) | 135 (68.88%) |  |
| Other Gender Identity | 5 (0.89%) | 3 (0.82%) | 2 (1.02%) |  |
| Sexuality |  |  |  | 0.105 |
| Heterosexual | 447 (81.27%) | 284 (80.00%) | 163 (83.59%) |  |
| I prefer not to answer | 75 (13.64%) | 56 (15.77%) | 19 (9.74%) |  |
| Other - Please specify | 8 (1.45%) | 4 (1.13%) | 4 (2.05%) |  |
| Bisexual | 11 (2.00%) | 5 (1.41%) | 6 (3.08%) |  |
| Homosexual (gay or lesbian) | 9 (1.64%) | 6 (1.69%) | 3 (1.54%) |  |
| Martial\_Status |  |  |  | 0.080 |
| I prefer not to say | 5 (0.89%) | 3 (0.82%) | 2 (1.02%) |  |
| Married or union | 252 (44.92%) | 164 (44.93%) | 88 (44.90%) |  |
| Other | 4 (0.71%) | 3 (0.82%) | 1 (0.51%) |  |
| Separated or divorced | 57 (10.16%) | 37 (10.14%) | 20 (10.20%) |  |
| Single | 208 (37.08%) | 140 (38.36%) | 68 (34.69%) |  |
| Widowed/widowed | 35 (6.24%) | 18 (4.93%) | 17 (8.67%) |  |
| Age | 43.89 (14.03) | 42.77 (14.10) | 45.98 (13.70) | 0.108 |
| Ethnicity |  |  |  | 0.084 |
| Hispanico | 143 (34.13%) | 88 (32.96%) | 55 (36.18%) |  |
| I prefer not to answer | 51 (12.17%) | 37 (13.86%) | 14 (9.21%) |  |
| Indigena | 86 (20.53%) | 51 (19.10%) | 35 (23.03%) |  |
| None - Does not identify with an ethnic group | 133 (31.74%) | 87 (32.58%) | 46 (30.26%) |  |
| Other | 6 (1.43%) | 4 (1.50%) | 2 (1.32%) |  |
| Religion |  |  |  | 0.093 |
| Catholic | 279 (50.00%) | 194 (53.30%) | 85 (43.81%) |  |
| Other | 22 (3.94%) | 14 (3.85%) | 8 (4.12%) |  |
| None | 57 (10.22%) | 36 (9.89%) | 21 (10.82%) |  |
| Christian or Evangelical | 200 (35.84%) | 120 (32.97%) | 80 (41.24%) |  |
| Education |  |  |  | 0.098 |
| Baccalaureate | 223 (39.96%) | 146 (40.22%) | 77 (39.49%) |  |
| Higher Education | 22 (3.94%) | 15 (4.13%) | 7 (3.59%) |  |
| None | 14 (2.51%) | 6 (1.65%) | 8 (4.10%) |  |
| Other - Please specify | 4 (0.72%) | 2 (0.55%) | 2 (1.03%) |  |
| Preschool | 9 (1.61%) | 6 (1.65%) | 3 (1.54%) |  |
| Primary | 150 (26.88%) | 95 (26.17%) | 55 (28.21%) |  |
| Professional Technical Level | 30 (5.38%) | 22 (6.06%) | 8 (4.10%) |  |
| Technical | 77 (13.80%) | 50 (13.77%) | 27 (13.85%) |  |
| University undergraduate | 29 (5.20%) | 21 (5.79%) | 8 (4.10%) |  |
| Economic Status |  |  |  | 0.085 |
| Stratum 1-2 (Very Low - Low) | 546 (98.20%) | 358 (99.17%) | 188 (96.41%) |  |
| Stratum 3-4 (Medium Low - Medium) | 10 (1.80%) | 3 (0.83%) | 7 (3.59%) |  |
| Area of Residence |  |  |  | 0.040 |
| Rural | 167 (29.93%) | 103 (28.45%) | 64 (32.65%) |  |
| Urban | 391 (70.07%) | 259 (71.55%) | 132 (67.35%) |  |
| Victim\_Registry |  |  |  | 0.076 |
| I am not registered because I am not a victim | 23 (4.11%) | 11 (3.02%) | 12 (6.15%) |  |
| I am not registered but I am a victim | 53 (9.48%) | 34 (9.34%) | 19 (9.74%) |  |
| Yes, I am registered | 483 (86.40%) | 319 (87.64%) | 164 (84.10%) |  |
| Were you or a close family member (e.g. sibling or child) forcibly recruited to be part of the revolutionary armed forces? | 220 (39.64%) | 132 (36.67%) | 88 (45.13%) | 0.079 |
|  |  |  |  |  |
| --- | --- | --- | --- | --- |
| 1 n (%); Mean (SD) | | | | |

```
#  gt::gtsave(as_gt(table1), filename = "Demographic_table.rtf")

data |> 
rstatix::wilcox_test(dem3 ~ pclgroup, alternative = "two.sided")
## # A tibble: 1 × 7
##   .y.   group1 group2    n1    n2 statistic      p
## * <chr> <chr>  <chr>  <int> <int>     <dbl>  <dbl>
## 1 dem3  0      1        365   196     31087 0.0105
```

```
options(scipen=10, digits = 4)

table2a <-
data |> 
  mutate(Assignment      = factor(pclgroup, labels = c("Control", "Case")),
         Gender          = dem2.factor,
         Sexuality       = dem6.factor,
         Age             = dem3,
         Ethnicity       = dem11.factor,
         Religion        = dem12.factor,
         Education       = dem14.factor,
         Economic_Stat   = dem17.factor,
         Living_Area     = dem8.factor,
         Victim_Registry = vic1.factor,
         FARC_Involved   = farc.factor)  |>  
  select(Assignment, ntrauma, lec1.factor, lec2a.factor, lec3a.factor, lec4a.factor,
         lec5a.factor, lec6a.factor, lec7a.factor, lec8a.factor,
         lec9a.factor, lec10a.factor, lec11a.factor, lec12a.factor,
         lec13a.factor, lec14a.factor, lec15a.factor, lec16a.factor,
         lec17a.factor, lec18a.factor, lec19a.factor, lec20a.factor,
         lec21a.factor, lec22a.factor) |> 
  tbl_summary(by = Assignment,
    label = list(lec1.factor = "Natural Disaster", lec2a.factor = "Fire or Explosion",
                 lec3a.factor = "Traffic Accident", lec4a.factor = "Serious Accident", 
                 lec5a.factor = "Exposure to Toxic Substances", lec6a.factor = "Physical Assault", 
                 lec7a.factor = "Armed Assault", lec8a.factor = "Sexual Assault",
                 lec9a.factor = "Unwanted Sexual Contact", lec10a.factor = "Combat", 
                 lec11a.factor = "Captivity", lec12a.factor = "Life Threatening Illness or Injury",
                 lec13a.factor = "Severe Human Suffering", lec14a.factor = "Sudden Violent Death", 
                 lec15a.factor = "Sudden Accidental Death", lec16a.factor = "Serious Injury, Harm, or Death Caused to Another",
                 lec17a.factor = "Parent or Partner Ridicule", lec18a.factor = "Physical Torture", 
                 lec19a.factor = "Psychological Torture", lec20a.factor = "House or Property Damaged",
                 lec21a.factor = "Forced Displacement", lec22a.factor = "Other Stressful Event",
                 worsttrauma.factor = "Index Trauma"),
    statistic = all_continuous() ~ "{mean} ({sd})",
    digits = list(all_continuous() ~ 2, all_categorical() ~ c(0, 2)),
    missing = "no") |> 
    add_overall() |> 
    add_p(list(all_categorical() ~ "chisq.test",
               all_continuous()  ~ "wilcox.test"),  include = everything()) |> 
    modify_header(statistic ~ "**Test Statistic**")   |> 
  add_stat(fns = list(all_continuous()  ~ Eff_size_r,
                      all_categorical() ~ Eff_size_V)) |> 
  modify_header(add_stat_1 ~ "**Effect Size**"); table2a
```

| **Characteristic** | **Overall**  N = 5621 | **Control**  N = 3661 | **Case**  N = 1961 | **Test Statistic**2 | **p-value**2 | **Effect Size** |
| --- | --- | --- | --- | --- | --- | --- |
| ntrauma | 8.23 (4.19) | 7.22 (3.85) | 10.10 (4.17) | 21,853 | <0.001 | 0.323 |
| Natural Disaster | 207 (36.83%) | 128 (34.97%) | 79 (40.31%) | 1.34 | 0.2 | 0.049 |
| Fire or Explosion | 199 (35.41%) | 117 (31.97%) | 82 (41.84%) | 5.01 | 0.025 | 0.094 |
| Traffic Accident | 196 (34.88%) | 119 (32.51%) | 77 (39.29%) | 2.29 | 0.13 | 0.064 |
| Serious Accident | 221 (39.32%) | 130 (35.52%) | 91 (46.43%) | 5.92 | 0.015 | 0.103 |
| Exposure to Toxic Substances | 96 (17.08%) | 62 (16.94%) | 34 (17.35%) | 0.000 | >0.9 | 0.000 |
| Physical Assault | 291 (51.78%) | 174 (47.54%) | 117 (59.69%) | 7.07 | 0.008 | 0.112 |
| Armed Assault | 249 (44.31%) | 142 (38.80%) | 107 (54.59%) | 12.3 | <0.001 | 0.148 |
| Sexual Assault | 97 (17.26%) | 53 (14.48%) | 44 (22.45%) | 5.13 | 0.024 | 0.096 |
| Unwanted Sexual Contact | 74 (13.17%) | 39 (10.66%) | 35 (17.86%) | 5.18 | 0.023 | 0.096 |
| Combat | 298 (53.02%) | 180 (49.18%) | 118 (60.20%) | 5.79 | 0.016 | 0.102 |
| Captivity |  |  |  | 12.6 | 0.002 | 0.150 |
| No | 323 (57.47%) | 226 (61.75%) | 97 (49.49%) |  |  |  |
| Yes, it happened to a family member | 172 (30.60%) | 108 (29.51%) | 64 (32.65%) |  |  |  |
| Yes, it happened to me | 67 (11.92%) | 32 (8.74%) | 35 (17.86%) |  |  |  |
| Life Threatening Illness or Injury | 137 (24.38%) | 62 (16.94%) | 75 (38.27%) | 30.3 | <0.001 | 0.232 |
| Severe Human Suffering | 303 (53.91%) | 167 (45.63%) | 136 (69.39%) | 28.1 | <0.001 | 0.223 |
| Sudden Violent Death | 236 (41.99%) | 119 (32.51%) | 117 (59.69%) | 37.6 | <0.001 | 0.259 |
| Sudden Accidental Death | 143 (25.44%) | 73 (19.95%) | 70 (35.71%) | 15.9 | <0.001 | 0.168 |
| Serious Injury, Harm, or Death Caused to Another | 21 (3.74%) | 6 (1.64%) | 15 (7.65%) | 11.2 | <0.001 | 0.141 |
| Parent or Partner Ridicule | 202 (35.94%) | 103 (28.14%) | 99 (50.51%) | 26.8 | <0.001 | 0.218 |
| Physical Torture | 101 (17.97%) | 43 (11.75%) | 58 (29.59%) | 26.4 | <0.001 | 0.217 |
| Psychological Torture | 253 (45.02%) | 137 (37.43%) | 116 (59.18%) | 23.5 | <0.001 | 0.205 |
| House or Property Damaged | 188 (33.45%) | 111 (30.33%) | 77 (39.29%) | 4.21 | 0.040 | 0.087 |
| Forced Displacement | 498 (88.61%) | 328 (89.62%) | 170 (86.73%) | 0.785 | 0.4 | 0.037 |
| Other Stressful Event | 203 (36.12%) | 103 (28.14%) | 100 (51.02%) | 28.0 | <0.001 | 0.223 |
|  |  |  |  |  |  |  |
| --- | --- | --- | --- | --- | --- | --- |
| 1 Mean (SD); n (%) | | | | | | |
| 2 Wilcoxon rank sum test; Pearson’s Chi-squared test | | | | | | |

```
#  gt::gtsave(as_gt(table2a), filename = "TraumaEndorsement_table.rtf")


table2b <-
data |> 
  mutate(Assignment      = factor(pclgroup, labels = c("Control", "Case")),
         Gender          = dem2.factor,
         Sexuality       = dem6.factor,
         Age             = dem3,
         Ethnicity       = dem11.factor,
         Religion        = dem12.factor,
         Education       = dem14.factor,
         Economic_Stat   = dem17.factor,
         Living_Area     = dem8.factor,
         Victim_Registry = vic1.factor,
         FARC_Involved   = farc.factor,
         worsttrauma.factor = factor(worsttrauma.factor))  |>  
  select(Assignment, worsttrauma.factor) |> 
  tbl_summary(by = Assignment,
    label = list(worsttrauma.factor = "Index Trauma"),
    statistic = all_continuous() ~ "{mean} ({sd})",
    digits = list(all_continuous() ~ 2, all_categorical() ~ c(0, 2)),
    missing = "no") |> 
    add_overall(); table2b
```

| **Characteristic** | **Overall**  N = 5621 | **Control**  N = 3661 | **Case**  N = 1961 |
| --- | --- | --- | --- |
| Index Trauma |  |  |  |
| A life-threatening illness or injury | 4 (0.74%) | 3 (0.87%) | 1 (0.52%) |
| A natural disaster (such as earthquakes, floods, volcanic eruptions, hurricanes, landslides) | 18 (3.35%) | 15 (4.35%) | 3 (1.55%) |
| Accident at work, at home or during recreational activities | 8 (1.49%) | 2 (0.58%) | 6 (3.11%) |
| Another stressful experience or event | 6 (1.12%) | 4 (1.16%) | 2 (1.04%) |
| Any other type of unwanted or unpleasant sexual activity | 5 (0.93%) | 3 (0.87%) | 2 (1.04%) |
| Armed assault (he has been shot, stabbed , threatened with a knife. Firearm or bomb) | 17 (3.16%) | 6 (1.74%) | 11 (5.70%) |
| Captivity (kidnapping , hostage-taking, prisoner of war, being deprived of liberty) | 12 (2.23%) | 7 (2.03%) | 5 (2.59%) |
| Combat or exposure to a war zone (such as military, combatant, or civilian) | 29 (5.39%) | 18 (5.22%) | 11 (5.70%) |
| Exposure to toxic substances | 2 (0.37%) | 2 (0.58%) | 0 (0.00%) |
| Fire, Explosion, or Bombs | 58 (10.78%) | 45 (13.04%) | 13 (6.74%) |
| Forced displacement | 233 (43.31%) | 163 (47.25%) | 70 (36.27%) |
| House or property damaged | 1 (0.19%) | 1 (0.29%) | 0 (0.00%) |
| Physical assault (attack, robbery, beating, has been kicked or beaten) | 13 (2.42%) | 10 (2.90%) | 3 (1.55%) |
| Physical torture | 2 (0.37%) | 1 (0.29%) | 1 (0.52%) |
| Psychological torture | 12 (2.23%) | 6 (1.74%) | 6 (3.11%) |
| Severe suffering | 10 (1.86%) | 4 (1.16%) | 6 (3.11%) |
| Sexual assault (rape, attempted rape, forced to engage in any sexual act with use of force or threats ) | 20 (3.72%) | 9 (2.61%) | 11 (5.70%) |
| Traffic accident (includes accident of car, bus, boat or boat, train or plane ) | 14 (2.60%) | 6 (1.74%) | 8 (4.15%) |
| Unexpected accidental death | 7 (1.30%) | 5 (1.45%) | 2 (1.04%) |
| Violent death and unexpected (murder, suicide) | 57 (10.59%) | 30 (8.70%) | 27 (13.99%) |
| Your parents, partner or family member ridiculed repeatedly, verbally abused, or said you re not worth it | 10 (1.86%) | 5 (1.45%) | 5 (2.59%) |
|  |  |  |  |
| --- | --- | --- | --- |
| 1 n (%) | | | |

```
#  gt::gtsave(as_gt(table2b), filename = "IndexTrauma_table.rtf")
```

```
table3 <-
data |> 
  mutate(Assignment  = factor(pclgroup, labels = c("Control", "Case")),
         Tot_ACES    = as.numeric(ace1+ace2+ace3+ace4+ace5+ace6+ace7+ace8+ace9+ace10, label = "ACE Score Total"),
         Tot_Dep     = as.numeric(phq1+phq2+phq3+phq4+phq5+phq6+phq7+phq8+phq9, label = "Depression Total"),
         Cut_Dep     = if_else(Tot_Dep >= 10, true = 1, false = 0, missing = NULL),
         Tot_Anx     = as.numeric(gad1+gad2+gad3+gad4+gad5+gad6+gad7, label = "Anxiety Total"),
         Cut_Anx     = if_else(Tot_Anx >= 10, true = 1, false = 0, missing = NULL),
         Tot_AUDIT   = as.numeric(audit1+audit2+audit3+audit4+audit5+audit6+audit7+audit8+audit9+audit10, label = "AUDIT Total"),
         Cut_AUDIT   = if_else(Tot_AUDIT >= 10, true = 1, false = 0, missing = NULL)) |> 
    rowwise() |> 
    mutate(N_disorder  = sum(pclgroup, Cut_Dep, Cut_Anx, Cut_AUDIT, na.rm = TRUE),
           Diss_Exp    = mean(c(des1,des2,des3,des4,des5,des6,des7,des8), na.rm = TRUE)) |> 
    ungroup() |>  
  select(Assignment, Tot_Dep, Cut_Dep, Tot_Anx, Cut_Anx, Cut_AUDIT, N_disorder, Diss_Exp) |> 
  tbl_summary(by = Assignment,
              label = list(Tot_Dep ~ "Total Depression Score",
                           Cut_Dep ~ "Probable Depression (>= 10)",
                           Tot_Anx ~ "Total Anxiety Score",
                           Cut_Anx ~ "Probable Anxiety (>= 10)",
                           Cut_AUDIT ~ "Probable Alcohol Use Disorder (>= 10)",
                           Diss_Exp ~ "Dissociative Experiences",
                           N_disorder ~ "Number of Probable Common Mental Health Diagnoses (0-4)"),
    type = list(Tot_Dep ~ "continuous", 
                Cut_Dep ~ "dichotomous",
                Tot_Anx ~ "continuous",
                Cut_Anx ~ "dichotomous",
                N_disorder ~ "continuous"),
    statistic = all_continuous() ~ "{mean} ({sd})",
    digits = list(all_continuous() ~ 2, all_categorical() ~ c(0, 2)),
    missing = "no") |> 
    add_overall() |> 
    add_p(list(all_categorical() ~ "chisq.test",
               all_continuous() ~ "wilcox.test"),  include = everything()) |> 
    modify_header(statistic ~ "**Test Statistic**") |> 
  add_stat(fns = list(all_continuous()  ~ Eff_size_r,
                      all_categorical() ~ Eff_size_V)) |> 
  modify_header(add_stat_1 ~ "**Effect Size**") |> 
    modify_caption("**Trauma and Diagnostic Characteristics**"); table3
```

**Trauma and Diagnostic Characteristics**

| **Characteristic** | **Overall**  N = 5621 | **Control**  N = 3661 | **Case**  N = 1961 | **Test Statistic**2 | **p-value**2 | **Effect Size** |
| --- | --- | --- | --- | --- | --- | --- |
| Total Depression Score | 6.96 (6.76) | 4.12 (4.84) | 12.01 (6.77) | 10,233 | <0.001 | 0.566 |
| Probable Depression (>= 10) | 157 (29.90%) | 43 (12.80%) | 114 (60.32%) | 128 | <0.001 | 0.494 |
| Total Anxiety Score | 6.60 (5.89) | 4.13 (4.48) | 11.15 (5.45) | 10,079 | <0.001 | 0.576 |
| Probable Anxiety (>= 10) | 148 (27.56%) | 44 (12.64%) | 104 (55.03%) | 108 | <0.001 | 0.449 |
| Probable Alcohol Use Disorder (>= 10) | 22 (13.66%) | 11 (9.82%) | 11 (22.45%) | 3.60 | 0.058 | 0.150 |
| Number of Probable Common Mental Health Diagnoses (0-4) | 0.93 (1.16) | 0.27 (0.59) | 2.17 (0.92) | 3,668 | <0.001 | 0.806 |
| Dissociative Experiences | 1.07 (1.84) | 0.88 (1.70) | 1.43 (2.02) | 26,943 | <0.001 | 0.179 |
|  |  |  |  |  |  |  |
| --- | --- | --- | --- | --- | --- | --- |
| 1 Mean (SD); n (%) | | | | | | |
| 2 Wilcoxon rank sum test; Pearson’s Chi-squared test | | | | | | |

```
#  gt::gtsave(as_gt(table3), filename = "Diagnoses_Table.rtf")


  data1 <- data |> 
  mutate(Assignment  = factor(pclgroup, labels = c("Control", "Case")),
         Tot_ACES    = as.numeric(ace1+ace2+ace3+ace4+ace5+ace6+ace7+ace8+ace9+ace10, label = "ACE Score Total"),
         Tot_Dep     = as.numeric(phq1+phq2+phq3+phq4+phq5+phq6+phq7+phq8+phq9, label = "Depression Total"),
         Cut_Dep     = if_else(Tot_Dep >= 10, true = 1, false = 0, missing = NULL),
         Tot_Anx     = as.numeric(gad1+gad2+gad3+gad4+gad5+gad6+gad7, label = "Anxiety Total"),
         Cut_Anx     = if_else(Tot_Anx >= 10, true = 1, false = 0, missing = NULL),
         Tot_AUDIT   = as.numeric(audit1+audit2+audit3+audit4+audit5+audit6+audit7+audit8+audit9+audit10, label = "AUDIT Total"),
         Cut_AUDIT   = if_else(Tot_AUDIT >= 10, true = 1, false = 0, missing = NULL)) |> 
    rowwise() |> 
    mutate(N_disorder  = sum(pclgroup, Cut_Dep, Cut_Anx, Cut_AUDIT, na.rm = TRUE)) |> 
    ungroup()

  
  
  data1 |> dplyr::select(N_disorder) |> 
    dplyr::mutate(Any_disorder = N_disorder >= 1) |> 
    tbl_summary(digits = list(all_continuous() ~ 2, all_categorical() ~ c(0, 2)),
                label = list(N_disorder ~ "Number of Probable Common Mental Health Diagnoses (0-4)",
                             Any_disorder ~ "Any Probable Diagnosis (Depression, Anxiety, PTSD, Alcohol Use)"),) |> 
    modify_caption("**Number of Participants With Any Probable Diagnosis, and Comorbidity Count**")
```

**Number of Participants With Any Probable Diagnosis, and Comorbidity Count**

| **Characteristic** | **N = 562**1 |
| --- | --- |
| Number of Probable Common Mental Health Diagnoses (0-4) |  |
| 0 | 294 (52.31%) |
| 1 | 111 (19.75%) |
| 2 | 64 (11.39%) |
| 3 | 88 (15.66%) |
| 4 | 5 (0.89%) |
| Any Probable Diagnosis (Depression, Anxiety, PTSD, Alcohol Use) | 268 (47.69%) |
|  |  |
| --- | --- |
| 1 n (%) | |

## Missing Data Analysis

```
data1 |> 
    select(pcl_1:pcl_20, audit1, phq1:phq9, gad1:gad7, des1:des8,
         dem2, dem6, dem4, dem3, dem11,
         dem12, dem14, dem17, dem8, vic1, farc,
         lec1, lec2a, lec3a, lec4a, lec5a, lec6a, lec7a,
         lec8a, lec9a, lec10a, lec11a, lec12a, lec13a, lec14a, lec15a, lec16a,
         lec17a, lec18a, lec19a, lec20a, lec21a, lec22a) |> 
  naniar::pct_miss()
## [1] 1.323

miss_dataplot <- data1 |> 
      select(pcl_1:pcl_20, N_disorder, phq1:phq9, gad1:gad7,
           dem2, dem6, dem4, dem3, dem11,
           dem12, dem14, dem17, dem8, vic1, farc,
           lec1, lec2a, lec3a, lec4a, lec5a, lec6a, lec7a,
           lec8a, lec9a, lec10a, lec11a, lec12a, lec13a, lec14a, lec15a, lec16a,
           lec17a, lec18a, lec19a, lec20a, lec21a, lec22a) |> 
  naniar::vis_miss(); miss_dataplot
```

```
MCAR_test <- data1 |> 
    select(pcl_1:pcl_20, audit1, phq1:phq9, gad1:gad7, des1:des8,
         dem2, dem6, dem4, dem3, dem11,
         dem12, dem14, dem17, dem8, vic1, farc,
         lec1, lec2a, lec3a, lec4a, lec5a, lec6a, lec7a,
         lec8a, lec9a, lec10a, lec11a, lec12a, lec13a, lec14a, lec15a, lec16a,
         lec17a, lec18a, lec19a, lec20a, lec21a, lec22a
         ) |> 
  naniar::mcar_test(); MCAR_test
## # A tibble: 1 × 4
##   statistic    df p.value missing.patterns
##       <dbl> <dbl>   <dbl>            <int>
## 1     6408.  7228    1.00               99
```

# Figure 1

```
library(patchwork)
cbpal <- c("#0072b2", "#009e73")

fig1a <- data |> 
ggplot(aes(x = "", y = pclscore)) +
geom_violin(alpha = 0.3, fill = "#13315C") +  
geom_boxplot(alpha = 0.6, width = .4, outlier.shape = NA, color = "#13315C") +
  expand_limits(y = c(0, 80)) +
geom_hline(yintercept = 33, linetype="dashed", color = "#ef8a62", size = .9) + 
theme_minimal() + theme(legend.position="none", axis.line = element_line(colour = "grey50", size = 1)) +  
          labs(title=NULL, x = "Total Sample", y = NULL) + coord_flip()

fig1b <- data |> 
mutate(pclgroup = factor(pclgroup, labels = c("Control", "Case"))) |> 
ggplot(aes(x = pclgroup, y = pclscore, color = pclgroup, fill = pclgroup)) +
geom_violin(alpha = 0.3) +  
geom_boxplot(alpha = 0.6, width = .4, outlier.shape = NA) +  
    expand_limits(y = c(0, 80)) +
scale_color_manual(values = cbpal) + scale_fill_manual(values = cbpal) +
theme_minimal() + theme(legend.position="none", axis.line = element_line(colour = "grey50", size = 1)) + 
          labs(title=NULL, x = "Assignment", y = "PTSD Score") + coord_flip()

jpeg("Figure 1.jpeg", width=1200, height=480)
fig1 <- fig1a / fig1b
fig1 + plot_annotation(caption = "Note: Cut-off score of 33 used to indicated probable PTSD diagnosis (Murphy et al., 2017)")
dev.off()
## png 
##   2
```
